# Structure-Based Design of Head-Only Fusion Glycoprotein Immunogens for Respiratory Syncytial Virus

**DOI:** 10.1371/journal.pone.0159709

**Published:** 2016-07-27

**Authors:** Jeffrey C. Boyington, M. Gordon Joyce, Mallika Sastry, Guillaume B. E. Stewart-Jones, Man Chen, Wing-Pui Kong, Joan O. Ngwuta, Paul V. Thomas, Yaroslav Tsybovsky, Yongping Yang, Baoshan Zhang, Lei Chen, Aliaksandr Druz, Ivelin S. Georgiev, Kiyoon Ko, Tongqing Zhou, John R. Mascola, Barney S. Graham, Peter D. Kwong

**Affiliations:** 1 Vaccine Research Center, National Institute of Allergy and Infectious Diseases, National Institutes of Health, Bethesda, Maryland, 20892, United States of America; 2 Electron Microscopy Laboratory, Cancer Research Technology Program, Leidos Biomedical Research, Inc., Frederick National Laboratory for Cancer Research, Frederick, Maryland, United States of America; University of Iowa, UNITED STATES

## Abstract

Respiratory syncytial virus (RSV) is a significant cause of severe respiratory illness worldwide, particularly in infants, young children, and the elderly. Although no licensed vaccine is currently available, an engineered version of the metastable RSV fusion (F) surface glycoprotein—stabilized in the pre-fusion (pre-F) conformation by “DS-Cav1” mutations—elicits high titer RSV-neutralizing responses. Moreover, pre-F-specific antibodies, often against the neutralization-sensitive antigenic site Ø in the membrane-distal head region of trimeric F glycoprotein, comprise a substantial portion of the human response to natural RSV infection. To focus the vaccine-elicited response to antigenic site Ø, we designed a series of RSV F immunogens that comprised the membrane-distal head of the F glycoprotein in its pre-F conformation. These “head-only” immunogens formed monomers, dimers, and trimers. Antigenic analysis revealed that a majority of the 70 engineered head-only immunogens displayed reactivity to site Ø-targeting antibodies, which was similar to that of the parent RSV F DS-Cav1 trimers, often with increased thermostability. We evaluated four of these head-only immunogens in detail, probing their recognition by antibodies, their physical stability, structure, and immunogenicity. When tested in naïve mice, a head-only trimer, half the size of the parent RSV F trimer, induced RSV titers, which were statistically comparable to those induced by DS-Cav1. When used to boost DS-Cav1-primed mice, two head-only RSV F immunogens, a dimer and a trimer, boosted RSV-neutralizing titers to levels that were comparable to those boosted by DS-Cav1, although with higher site Ø-directed responses. Our results provide proof-of-concept for the ability of the smaller head-only RSV F immunogens to focus the vaccine-elicited response to antigenic site Ø. Decent primary immunogenicity, enhanced physical stability, potential ease of manufacture, and potent immunogenicity upon boosting suggest these head-only RSV F immunogens, engineered to retain the pre-fusion conformation, may have advantages as candidate RSV vaccines.

## Introduction

Human respiratory syncytial virus (RSV) infects most children by the age of two, with re-infection occurring sporadically throughout life [1]. Young children and the elderly are especially at risk of severe lower respiratory tract infection resulting in extensive morbidity and over 3.4 million hospitalizations per year [2–4]. The development of an RSV vaccine is thus a priority. To date, the most potently RSV-neutralizing antibodies recognize the RSV fusion (F) surface glycoprotein [5]. RSV F is a type I fusion glycoprotein, comprising an F_0_ precursor which is activated by furin cleavage to form a prefusion (pre-F) trimer of disulfide-linked F_2_ and F_1_ heterodimers [6]. The pre-F conformation of RSV F undergoes substantial structural rearrangements as it transitions to the post-fusion conformation, as part of its essential role in merging viral and host cell membranes during entry.

Among several antigenic sites on RSV F associated with neutralizing activity [7–12], antigenic site Ø, located at the membrane distal end of pre-F (head region), is recognized by several of the most potent RSV-neutralizing antibodies thus far identified [9, 13, 14] and is a major target of neutralizing antibodies elicited in RSV-infected humans [15]. As antigenic site Ø is only present on the pre-F conformation of RSV F, and recombinant pre-F spontaneously rearranges to the post-fusion conformation [9], several variants of RSV F stabilized in the pre-F form have been created for use as immunogens. DS-Cav1 [16], DS-Cav1-Cys-zipper [17], SC-TM [18] and SC-DM [18], all of which preserve antigenic site Ø, have been shown to elicit high titers of neutralizing antibodies in mice, cotton rats, and macaques. However, antigenic site Ø comprises less than 10% of the surface area of the RSV F trimer glycoprotein [9], providing ample opportunity for antigenic competition from irrelevant or less neutralization-sensitive epitopes on RSV F. A vaccine that were to more specifically present antigenic site Ø might thus have the potential to focus and to improve the quality of the antibody response.

Immune-focusing of the vaccine response, through the removal of extraneous epitopes from vaccine immunogens, has been observed to elicit targeted antibody responses in several studies. With RSV, incorporation of two RSV F helices from antigenic site II into a protein scaffold resulted in immunogens that could elicit binding antibodies to site II in mice [19] and—with the addition of an intervening loop—neutralizing antibodies to site II in macaques [20], though the macaque titers appeared to be much lower than those elicited by trimeric immunogens, such as the RSV F trimer. With influenza A virus, headless or stalk-only immunogens of the hemagglutinin (HA) glycoprotein induced significantly higher titers of protective HA stem-directed antibodies than the seasonal influenza trivalent inactivated vaccine in mice [21, 22] and ferrets [22], and HA stem-directed antibodies that reduced symptoms of disease in macaques [21]. Furthermore, with Middle East respiratory syndrome coronavirus, mice immunized with the receptor-binding domain (RBD)-containing S1 subunit elicited more RBD-reactive neutralizing antibodies than mice immunized with DNA for full-length spike (S) protein [23]. We therefore hypothesized that a “head-only” immunogen might more ably boost antigenic site Ø-directed responses than the complete F glycoprotein. To test this hypothesis, we designed a series of RSV F immunogens comprising only the membrane-distal half (head region) of the RSV F glycoprotein, with antigenic site Ø intact and the membrane-proximal half (stalk region) completely removed. Four “head-only” RSV F immunogens were evaluated in prime-boost mouse immunogenicity studies, with two of these boosting RSV-neutralization titers to levels that were comparable to those boosted by the RSV F DS-Cav1 trimer, although increased site Ø-directed antibodies.

## Materials and Methods

### Design of head-only RSV F immunogens

The amino acid sequence of RSV F subtype A, strain A2, with DS-Cav1 mutations S155C, S190F, V207L, and S290C [16], was used a starting point for each design. All designs included both a His-tag and a Strep-tag for use in purification and antibody-binding assays.

### Protein expression and purification

Head-only RSV F variants were expressed by transient transfection of Expi293F cells (Thermo Fisher Scientific, MA) using 293fectin (Thermo Fisher Scientific, MA). The culture supernatants were harvested 5 days post transfection and centrifuged at 10,000g to remove cell debris. The supernatants were sterile-filtered and the head only RSV F variants were purified by nickel and Strep-Tactin (iba, MO) affinity chromatography followed by size-exclusion chromatography (SEC) using a Superdex 200 16/60 column (GE Healthcare, PA). The purification tags were removed from the RSV F proteins by digestion with 1.0 U restriction-grade biotinylated thrombin (Novagen, WI) per mg of immunogen overnight at room temperature. Cleaved immunogens were subsequently passed over a streptavidin column (GE Healthcare, PA) to capture the thrombin and further purified by a second round of SEC in phosphate-buffered saline (PBS) prior to analysis or immunization. The molecular weights of proteins eluted from SEC were estimated by fitting elution volumes to a regression line in a plot of Log molecular weight versus (Ve-Vo)/(Vt-Vo) for several standard proteins where Ve is elution volume, Vo is the void volume of the column, and Vt is the total volume of the column.

### Expression and purification of antibodies and antigen-binding fragments (Fabs)

Antibodies were expressed by transient co-transfection of HEK 293-F cells (Thermo Fisher Scientific, MA) with heavy- and light-chain plasmids using 293fectin (Thermo Fisher Scientific, MA). Cell supernatants were harvested after 4–5 days and passed over Protein A agarose (GE Healthcare, PA). Bound antibodies were washed with PBS and eluted with IgG elution buffer (Pierce, IL) into 1/10th volume of 1 M Tris-HCl pH 8.0. Fabs were created by digesting the IgG with Lys-C or HRV3C protease [24], and the cleaved Fc region was removed by passing the mixture over Protein A agarose. Fabs were further purified by SEC.

### Antigenic screening of head-only RSV F immunogens

All constructs were assessed using a 96-well microplate format for high throughput expression followed by an ELISA-based antigenic evaluation as described previously [16]. Briefly, 24 h prior to transfection HEK 293T cells (Thermo Fisher Scientific, MA) were seeded in each well of a 96-well microplate at a density of 2.5x10^5^ cells/ml in expression medium (high glucose DMEM supplemented with 10% ultra-low IgG fetal bovine serum and 1x-non-essential amino acids), and incubated at 37°C, 5% CO_2_ for 20 h. Plasmid DNA and TrueFect-Max (United BioSystems, MD) were mixed and added to the growing cells, and the 96-well plate incubated at 37°C, 5% CO_2_. One day post transfection, enriched medium (high glucose DMEM plus 25% ultra-low IgG fetal bovine serum, 2x nonessential amino acids, 1x glutamine) was added to each well, and the 96-well plate was returned to the incubator for continuous culture. On day five post transfection, supernatants with the expressed RSV F variants were harvested and tested by ELISA for binding to D25 and motavizumab antibodies using Ni^2+^-NTA microplates. After incubating the harvested supernatants at 4°C for one week, two weeks and five weeks, ELISAs were repeated for recognition by antibodies D25, AM22 and 5C4 respectively.

### Antigenic characterization

A fortéBio Octet Red384 instrument (Pall ForteBio LLC, CA) was used to measure binding kinetics of RSV F head-only variants to antigenic site Ø (D25, AM22) and site II-targeting motavizumab antibodies. All assays were performed with agitation set to 1,000 rpm in PBS supplemented with 1% bovine serum albumin (BSA) in order to minimize nonspecific interactions. The final volume for all solutions was 50–80 μl/well. Assays were performed at 30°C in tilted black 384-well plates (Greiner Bio-One, NC). RSV F head-only variants (50 μg/ml) in PBS buffer was used to load anti-His-tag probes (HIS1K) for 300 s. Typical capture levels for each loading step were between 0.6 and 1.2 nm, and variability within a row of eight tips did not exceed 0.1 nm for each of these steps. The nm unit is a measure of the change in the interference pattern of white light reflected from the surface of the biosensor tip compared to an internal reference. This was measured in real-time and correlates with a change in the number of bound molecules on the biosensor tip surface. This can also be defined as a change in response units measured in nm [25, 26]. Biosensor tips were then equilibrated for 90 s in PBS + 1% BSA prior to measuring association with antigen binding fragments (Fabs) in solution (0.016 μM to 0.5 μM) for 300 s; Fabs were then allowed to dissociate for 300–1200 s. Parallel correction to subtract systematic baseline drift was carried out by subtracting the measurements recorded for a loaded sensor incubated in PBS + 1% BSA. Data analysis and curve fitting were carried out using Octet analysis software, version 8.0 (Pall ForteBio LLC, CA). Experimental data were fitted with a binding equation describing a 1:1 interaction. Global analyses of the complete data sets assuming reversible binding (full dissociation) were carried out using nonlinear least-squares fitting allowing a single set of binding parameters to be obtained simultaneously for all concentrations measured in a single experiment.

### Assessments of physical stability

To assess the physical stability of the pre-fusion conformation of RSV F head region proteins under various stress conditions, we treated the proteins with a variety of pharmaceutically relevant stresses such as extreme pH, high temperature, low and high osmolarity, and repeated freeze/thaw cycles [16, 17, 27]. The physical stability of treated RSV F head-only proteins was evaluated by retention of binding to the antigen-binding fragment (Fab) of the site Ø-specific antibody D25.

All treatments were carried out at a protein concentration of 100 μg/ml. For pH treatments, the RSV F glycoprotein solution was adjusted to either pH 3.5 with citrate buffer or pH 10 with *N*-cyclohexyl-3-aminopropanesulfonic acid (CAPS) buffer, incubated at room temperature for 60 minutes and subsequently neutralized to pH 7.5 with Tris buffer. Temperature treatments were carried out by incubating the RSV F glycoprotein solutions at 50°C, 70°C or 90°C for 60 minutes in a PCR cycler with heated lid. For osmolarity treatments, RSV F glycoprotein solutions originally containing 150 mM NaCl were either diluted with 2.5 mM Tris buffer (pH 7.5) to an osmolarity of 10 mM NaCl or adjusted with 4.5 M MgCl_2_ to a final concentration of 3.0 M MgCl_2_. Protein solutions were incubated for 60 minutes at room temperature and then returned to 150 mM salt by adding 5 M NaCl or dilution with 2.5 mM Tris buffer, respectively, and concentrated to 50 μg/ml. The freeze/thaw treatment was carried out by repeatedly freezing RSV F glycoprotein solutions in liquid nitrogen and thawing at 37°C ten times. All RSV F glycoproteins were diluted to 80 μg/ml with PBS + 1% BSA to reduce non-specific binding, and their ability to bind D25 Fab was measured with a fortéBio Octet instrument using the protocol described below.

All fortéBio assays were performed at 30°C in tilted black 384-well plates (Greiner Bio-One, NC) with agitation set to 1,000 rpm in and a well volume of 50 μl. Trimeric and dimeric RSV F head proteins i-210, i-447, and i-693 were immobilized using anti-Strep-tag IgG. Due to reduced avidity of the interaction between the anti-Strep-tag antibody and the monomeric RSV F head proteins, i-273 was immobilized using the higher affinity antigenic site II-directed motavizumab IgG. Pre-immobilized anti-mouse Fc biosensor tips were loaded with anti-Strep-tag (100 μg/ml in PBS + 0.2% BSA) for 600 s and pre-immobilized anti-human Fc biosensor tips were loaded with motavizumab IgG (50 μg/ml in PBS + 0.2% BSA) for 300 s. Variability in loading within a row of eight tips did not exceed 0.1 nm for each of these steps. Biosensor tips were equilibrated for 120 s in PBS + 0.2% BSA prior to loading RSV F variants for 300 s. Biosensor tips were then equilibrated for 120 s in PBS + 0.2% BSA prior to measuring association of D25 Fab (50 μg/ml in PBS + 0.2% BSA) for 300 s. Fabs were allowed to dissociate for 300 s in PBS + 0.2% BSA. Parallel correction to subtract systematic baseline drift was carried out by subtracting the measurements recorded for a loaded sensor incubated in PBS + 1% BSA. For trimeric RSV F head proteins i-210 and i-447, as well as monomeric RSV F head protein i-273, physical stability is reported as the ratio of steady state D25-binding level before and after stress treatment. Due to reduced avidity of the interaction between the anti-Strep-tag antibody and the dimeric RSV F head protein i-693, some dissociation of the antigen from the biosensor was observed over the 420 seconds of equilibration and D25 binding. To account for this, the physical stability of i-693 is reported as the ratio of maximum D25-binding level before and after stress treatment.

### Negative-stain electron microscopy

Samples were diluted with a buffer containing 20 mM HEPES, pH 7.0, and 150 mM NaCl, adsorbed to a freshly glow-discharged carbon-film grid for 15 s, washed with the above buffer, and stained with 0.7% uranyl formate. Images were collected at a magnification of 100,000 semi-automatically using SerialEM [28] on a FEI Tecnai T20 microscope operating at 200 kV and equipped with a 2k x 2k Eagle CCD camera. The pixel size was 0.22 nm/px. Particles were picked manually or using the swarm mode in e2boxer from the EMAN2 software package [29]. The datasets for i-693, i-693 + D25 complex, i-693 + motavizumab complex, i-693 + D25 + motavizumab complex, i-447, i-447 + D25 complex, i-447 + motavizumab complex, and i-447 + D25 + motavizumab complex contained 4872, 8915, 6354, 5847, 8806, 2242, 3031, and 6542 particles, respectively. The datasets for i-273, i-273 + D25 complex, i-273 + motavizumab complex, i-273 + D25 + motavizumab complex, i-210, i-210 + D25 complex, i-210 + motavizumab complex, and i-210 + D25 + motavizumab complex contained 3652, 2840, 4017, 4169, 2584, 2123, 1341, and 1939 particles, respectively. Initial reference-free 2D classification was performed using EMAN2. In cases where the quality of 2D classes was insufficient, classification was repeated using reference-free rotational alignment and classification (“AP C”) or reference-free alignment and correspondence analysis in SPIDER [30].

### Ethics statement

All animal experiments were reviewed and approved by the Animal Care and Use Committee of the Vaccine Research Center, NIAID, NIH, under animal protocol identification number 13–454, and all animals were housed and cared for in accordance with local, state, federal, and institute policies in an American Association for Accreditation of Laboratory Animal Care (AAALAC)-accredited facility at the NIH.

### Mouse immunizations

As with our prior experiments [16, 17], female hybrid mice that were the first filial offspring of a cross between BALB/cJ females (C) and C57BL/6J males (B6) (The Jackson Laboratory) known as CB6F1/J at ages 6 weeks to 12 weeks were intramuscularly injected with RSV F immunogens at week zero and week three for homologous prime-boosts and at week 10 for the final boost. Four groups (n = 10) of mice were initially primed with head-only immunogens and three groups (n = 10) were primed with DS-Cav1. The frozen RSV F variant immunogen proteins were thawed on ice and mixed with 5-fold w/w polyinosinic:polycytidylic acid (poly I:C) (Invivogen, CA) adjuvant (i.e. 10 μg immunogen, 50 μg Poly I:C in 100 μl per animal immunization), with injections taking place within one hour of immunogen:adjuvant preparation. Two injection sites were used for each immunization. No adverse effect from immunization was observed. Blood was collected at least three days before immunization, and at week two, week five, week seven and weeks 11 and 12 post initial immunization.

### Viruses and cells for neutralization assays

Viral stocks were prepared and maintained as previously described [31]. Recombinant mKate-RSV expressing prototypic subtype A (strain A2) F genes and the Katushka fluorescent protein were constructed as reported by Hotard et al. [32]. HEp-2 cells (ATCC, VA) were maintained in Eagle's minimal essential medium containing 10% fetal bovine serum (10% EMEM), supplemented with glutamine, penicillin and streptomycin.

### RSV neutralization and competition neutralization

Neutralization titers were measured by a fluorescence plate reader neutralization assay as described previously [15, 16]. Briefly, 2.4 x 10^4^ HEp-2 cells were seeded in 384-well black optical bottom plates (Nunc 384-well plates, Thermo Fisher Scientific, MA). Sera samples were assayed in four-fold dilutions from 1:10 to 1:40960, mixed with an equal volume of recombinant mKate-RSV expressing prototypic F genes from subtype A (strain A2) and the Katushka fluorescent protein, incubated at 37°C for one hour. Next, 50 μl diluted sample and virus were added to HEp-2 cells in 384-well assay plate, and incubated at 37°C for 22–26 hours. After incubation, fluorescence intensity was measured in a fluorescence plate reader at an excitation of 588 nm and an emission of 635 nm (SpectraMax Paradigm, Molecular Devices, CA). The IC_50_ for each sample was calculated by curve fitting and non-linear regression using GraphPad Prism (GraphPad Software Inc., CA). Sera neutralization titers elicited from the immunization prime followed by the first boost were measured once for each mouse. Neutralization titers elicited from the second boost were measured four times and averaged for each mouse, except for sera elicited from the final i-447 boost and from the 3×DS-Cav1 immunization, which were measured three times for each mouse before averaging.

As previously described [16], the protective neutralization threshold was calculated by noting that the clinical administration of palivizumab (Synagis) at 15 mg/kg, leads to patient sera levels at trough of ~40 μg/ml. This serum concentration provides protection in infants from severe disease and protection in cotton rats from RSV infection. In the neutralization assay described above, 40 μg/ml of palivizumab yields an EC_50_ of 100.

For the competition neutralization assay, 6 μg of probe (either DS-Cav-1, DS-Cav-1 antigenic site Ø knock out (KO) [15] or DS-Cav-1 antigenic site II KO [15]) were added to sera and incubated at 37°C for one hour followed by the addition of an equal volume of virus and incubation for an additional hour at 37°C. 50 μl of the sera-RSV F-RSV A2 mixture were added to 2.4 x10^4^ HEp-2 cells per well in a 384-well assay plate, and incubated at 37°C for 22–26 hours. The fluorescence intensity for each well was read as described above. One reading was obtained for each mouse sera sample.

### Sera binding analysis

A fortéBio Octet Red384 instrument was used to measure sera recognition of DS-Cav1, DS-Cav-1 antigenic site Ø KO [15] or DS-Cav-1 antigenic site II KO [15] probes. All assays were performed at 30°C in tilted black 384-well plates (Greiner Bio-One, NC) with agitation set to 1,000 rpm in PBS supplemented with 1% BSA and a well volume of 55 μl. DS-Cav1 and DS-Cav1 KO probes (50 μg/ml) were immobilized for 300 s on HIS1K biosensor tips and equilibrated for 60 s in PBS + 1% BSA prior to measuring association with sera diluted 1:200 in PBS supplemented with 1% BSA. The association after 300 s was recorded.

### Structural analysis

Surface area calculations were performed using Chimera [33] and the coordinates from PDB entry 4JHW [9]. RSV F epitope residues for D25 and motavizumab were delineated using the PISA web server [34] and the coordinates for PDB entries 4JHW [9] and 4ZYP [11] respectively. Structural figures were created using the program Chimera [33] and RSV F trimer coordinates from PDB entry 4JHW [9].

## Results

### Design strategies for head-only RSV F immunogens

**Fig 1:**
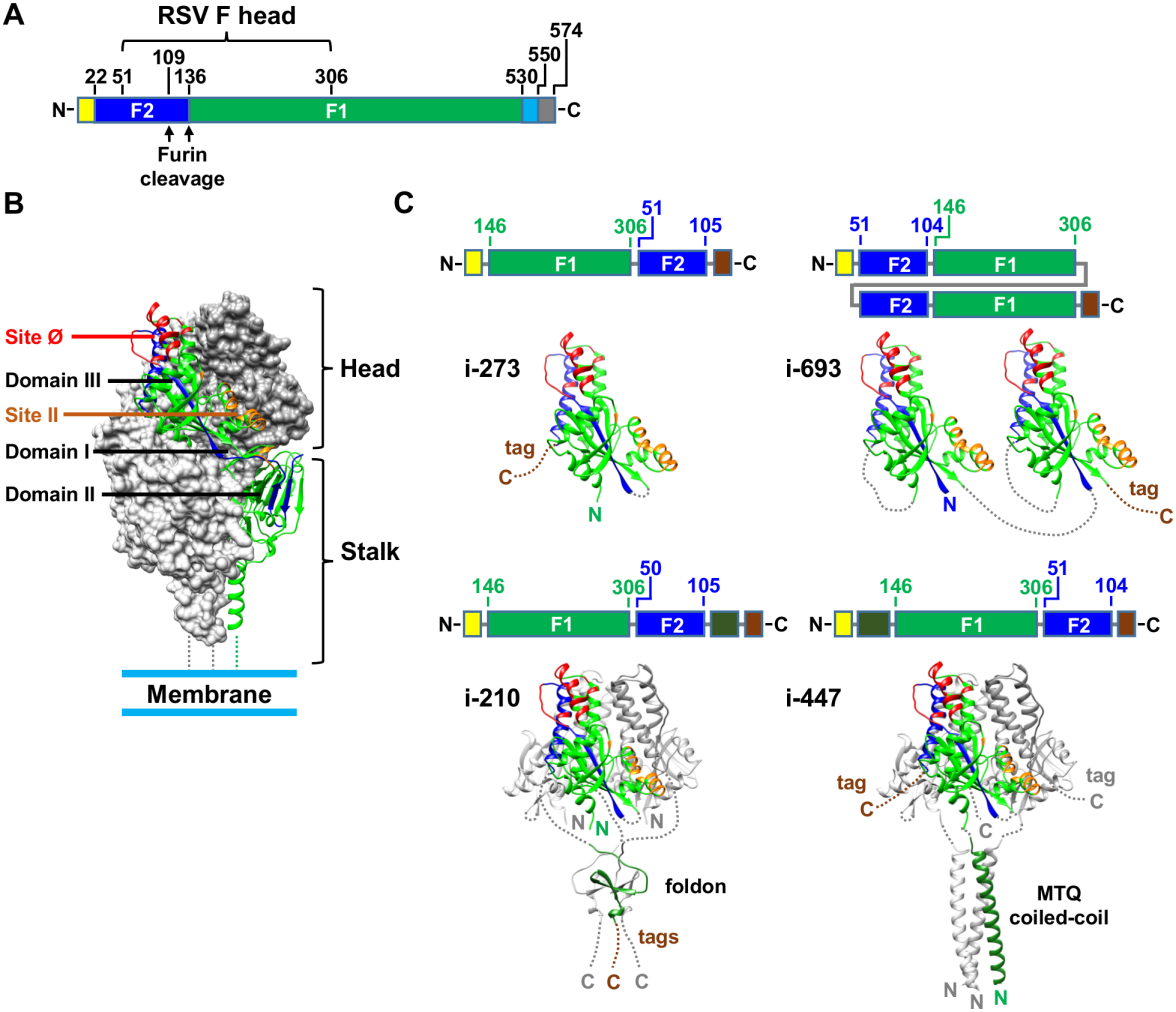
Design of RSV F head-only immunogens. **(A)** Genetic construct of RSV F illustrating the location of the head region. The signal peptide is colored yellow, F_2_ is colored blue, F_1_ is colored green, the transmembrane region is colored cyan and the cytoplasmic domain is colored gray. **(B)** Pre-F form of the RSV F trimer. One protomer of RSV F is depicted as a ribbon diagram and colored as in A. The other two protomers are gray surface representations. Antigenic site Ø and site II are red and orange respectively. Domains I, II and III are labeled as defined previously [9, 35]. **(C)** Models of head-only immunogens i-273, i-693 (upper panels), i-210 and i-447 (lower panels). For each immunogen a cartoon of the genetic construct is depicted (top) and a ribbon diagram (bottom), color-coded as in A and B with brown coloring for purification tags. PDB entries 4JHW [9], 1RFO [36] and 1GCM [37] were used to depict RSV F, the foldon and the coiled coil respectively. RSV F residue numbering follows the numbering in PDB entry 4JHW [9].

The pre-F structure of the RSV F trimeric glycoprotein can be divided into two halves: a membrane-proximal half, the RSV F “stalk” region, which comprises domains I and II and has a primarily β-strand structure that terminates in a C-terminal coiled coil that enters the membrane; and a membrane-distal half, the RSV F “head” region, which comprises domain III and is primarily α-helical (Fig 1A and 1B) [9]. These two halves are connected by a long, two-stranded β-sheet, containing one strand from F_1_ and another strand from F_2_. The RSV F head contains at least two epitopes associated with neutralizing antibodies.

Antigenic site Ø, located at the apex of the pre-F trimer, is recognized by potent neutralizing antibodies including D25, AM22 and 5C4 [9, 13, 14]. Antigenic site II, located towards the middle of the pre-F trimer (Fig 1B), is the binding site for antibodies palivizumab and motavizumab [9, 12, 38, 39], which are reported to be 10–100 fold less potent than site Ø-directed antibodies [16]. Antigenic sites I and IV, which are also recognized by antibodies with lower potency, are located on the RSV F stalk region [8, 9], and the epitopes for quaternary-specific antibodies, AM14 and MPE8, straddle both the head and stalk regions [10, 11].

Since the RSV F stalk comprises 55% of the total surface area of the RSV F trimer, we reasoned that expression of the head-only region of RSV F might help to focus the immune response to antigenic site Ø as well as to antigenic site II. We therefore designed 70 variants of head-only RSV F constructs using three different approaches (Fig 1C and S1 Table). For the first approach, domain III was expressed as a simple monomer (30 designs); in the second approach, domain III was expressed as a tandemly linked dimer (18 designs); in the third approach, a trimerization domain was fused to domain III to enable the expression of a trimeric protein resembling the native pre-fusion RSV F head (22 designs). The topology of RSV F is such that removal of the RSV F stalk from the RSV F head results in two separate F_1_ and F_2_ polypeptides within each head protomer. Reconnection of both polypeptides to make a single polypeptide afforded an opportunity to permute circularly the domain topology, by linking either F_2_ to F_1_, or F_1_ to F_2_ (S1 Fig). Most designs also incorporated the disulfide bond from DS-Cav1 (S155C-S290C) as well as the DS-Cav1 cavity filling mutations (S190F and V207L) [16] to stabilize the pre-fusion conformation. Numerous additional mutations included repacking of the protein interior, the addition of disulfide bonds, and the alteration of surface mutations to replace hydrophobic residues with hydrophilic residues or glycans. Moreover, various lengths of glycine rich linkers were used to link the two polypeptides of domain III and to connect them to trimerization domains. Finally, a signal peptide for secretion and a cleavable His-tag and Strep-tag to aid in purification were added to each construct.

### Antigenic characterization of 70 head-only RSV F immunogens

Designed constructs were codon optimized for expression in human cells, and transfected and expressed in HEK 293T cells by using a 96-well format, which coupled expression with evaluation of antigenic recognition [16]. As several of the most potently neutralizing RSV F antibodies target antigenic site Ø, three monoclonal antibodies (mAbs) specific for this site were used as readout: antibodies D25, 5C4 and AM22. Additionally, because soluble RSV F trimers are metastable and spontaneously convert to a post-fusion conformation, D25 binding was also evaluated after one week at 4°C, and AM22 and 5C4 binding were evaluated after two and five weeks, respectively, at 4°C. Out of 70 designs, 51 (73%) had D25 ELISA readouts of 1.5 OD_450_ or higher after one week at 4°C (S2–S4 Tables). To further gauge the stability of these immunogens, D25 recognition was also evaluated after one hour at 60, 70, 80, 90 and 100°C. Fifty of the head-only designs retained the majority of their D25 antigenicity after one hour at 70°C, conditions under which the parent DS-Cav1 RSV F trimer completely lost D25 recognition [16]. At 80°C and above, we observed a substantial decrease in D25 binding for most of the head-only immunogens (S2–S4 Tables).

### Top scoring monomeric, dimeric and trimeric head-only immunogens

To reduce the list of 50 immunogens stable at 70°C for further characterization, we calculated the average antigen score comprising the average of the maximum ELISA readings for binding D25 after one week at 4°C and after one hour at 70°C as well as for binding AM22 after two weeks at 4°C (S2–S4 Tables). The top scoring first approach construct was “i-273”, average antigenic score of 3.17, which was expected to be a monomer. The top scoring second approach construct was “i-693”, average antigenic score of 3.06, which was expected to be a dimer. The top scoring third approach constructs, expected to be trimers, were i-210 and i-447, each with average antigenic scores of 3.08.

Design i-273 was a circularly permutated domain III monomer (Fig 1C). The F_2_ segment (residues 51–105) was connected by a short GG linker to the C-terminal end of the F_1_ segment (146–306), which resulted in a different topology than the native RSV F (Fig 1C). In addition to the four mutations embodied in DS-Cav1, thirteen additional mutations (S2 Fig) were introduced. These included an additional disulfide bond between F_1_ and F_2_ as well as alteration of hydrophobic surface residues to hydrophilic glutamines to improve solubility.

Design i-693 was a tandemly linked dimer of two identical domain III monomers connected by a GSG linker (Fig 1C). Each monomer comprised F_2_ residues 51–105 linked to F_1_ residue 146–350 by a short GSG linker. Each monomer also contained the four DS-Cav1 mutations.

Design i-210 comprised a circularly permutated domain III monomer (F_1_ residues 146–306 connected to F_2_ residues 50–106 by a GGSGG linker), which was connected at the C-terminus of F_2_ by a GGSGGSG linker to a bacteriophage T4 foldon trimerization domain (Fig 1C). In addition to DS-Cav1 mutations, three hydrophobic surface residues were replaced by hydrophilic residues (S2 Fig).

Design i-447 was also a circularly permutated domain III monomer (F_1_ residues 146–306 connected to F_2_ residues 51–105 by a GGPG linker) (Fig 1C). However, in this case the trimerization domain was a computationally derived triple helical coiled coil referred to as MTQ [40] that was directly connected to the N-terminus of the domain III F_1_ polypeptide without a linker. Three mutations from DS-Cav1 (S190F, S155C and S290C) were included for stability.

### Physical characteristics of top scoring head-only immunogens

**Fig 2:**
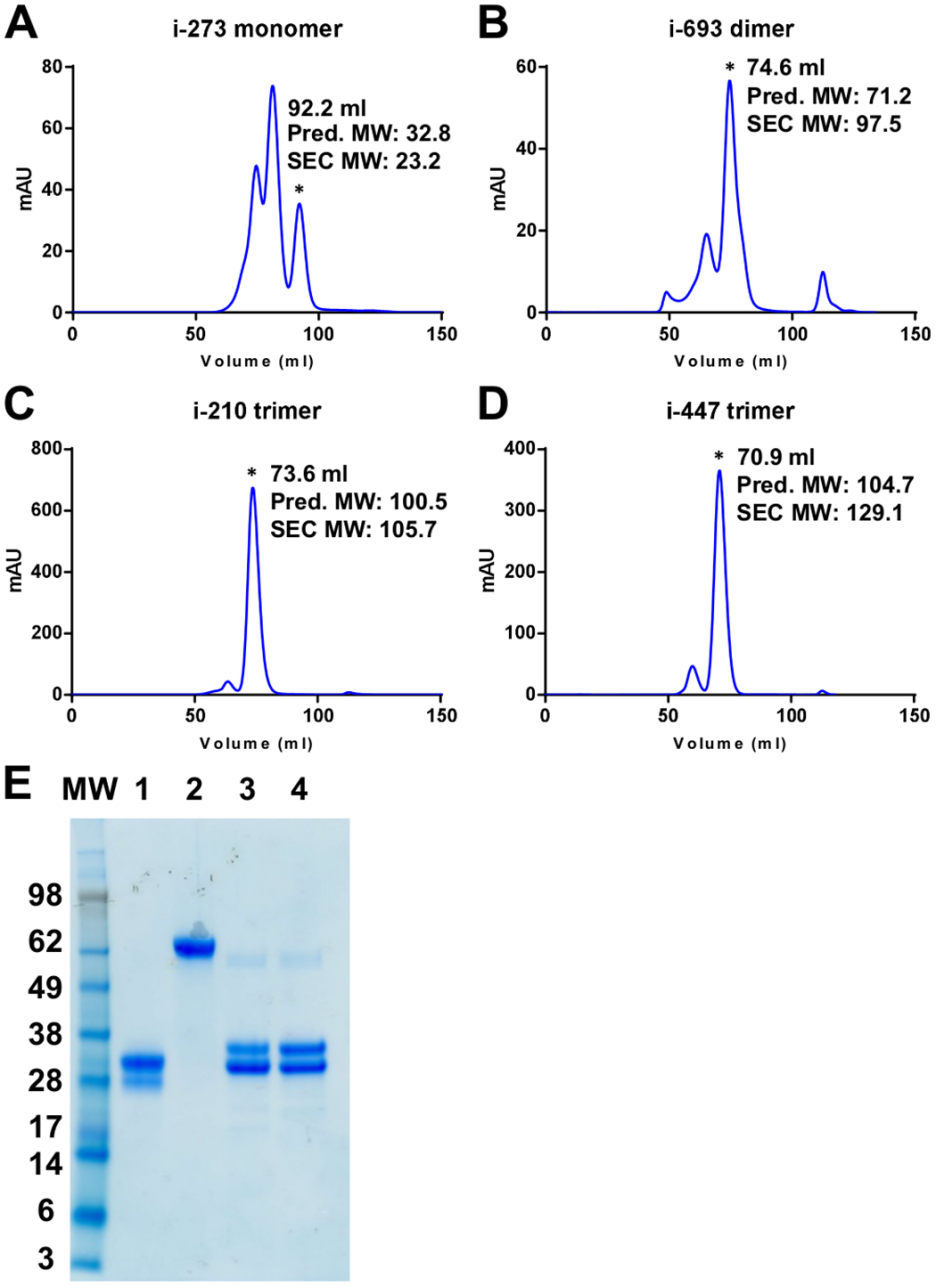
Characterization of head-only immunogens i-273, i-693, i-210 and i-447. **(A-D)** Size exclusion chromatography (SEC) analysis of all four immunogens after purification by affinity chromatography. For each immunogen an asterisk indicates the peak that was chosen for antigenic, physical, structural and immunization studies. The calculated molecular weight (MW) in kDa based on elution volume and the predicted MW based on the sequence and predicted glycan content (2.5 kDa/glycan) are also indicated. Immunogens i-273, i-693, i-210 and i-447 were predicted to have two, four, three and three glycans respectively based on the occurrence of NXT/S sequons in the sequence. **(E)** Reduced SDS-PAGE analysis (3μg/well) of immunogens (1) i-273, (2) i-693, (3) i-210 and (4) i-447 pooled from the asterisk-indicated peaks in (A-D). Doublet bands likely arise from differential glycosylation.

The four top scoring head-only immunogens were expressed in one liter cultures of Expi293F cells. After affinity purification and cleavage of purification tags, size exclusion chromatography was used to characterize their oligomeric states, and appropriate peaks pooled for further characterization. Design i-273 eluted as three peaks. Because it was designed as a monomer we chose the peak corresponding to the monomer for further characterization. Designs i-693, i-210, and i-447 eluted primarily as single peaks, corresponding to dimer, trimer and trimer, respectively (Fig 2).

We assessed these size-exclusion purified immunogens by biolayer interferometry for recognition by site Ø-directed antibodies D25 and AM22 as well as by the site II-directed antibody motavizumab. D25 and AM22 recognized all four immunogens with nanomolar affinity, though i-273 monomer and i-693 dimer displayed slightly lower affinity than the trimeric immunogens i-210 and i-447 (S3 Fig and Table 1). Although the antigenic site II-directed antibody motavizumab recognized both the i-447 trimer and the i-693 dimer with nanomolar affinity, the i-273 monomer and i-210 trimer each displayed only micromolar affinity. This was unsurprising as both i-273 and i-210 contained a L258K surface mutation in the middle of the motavizumab epitope and the immunogen with the lowest motavizumab affinity, i-273, had an additional epitope mutation of L273K (S2 Fig). Moreover, the four top scoring designs were chosen because of their recognition by site Ø-directed antibodies, with no consideration given to their recognition by site II-directed antibodies.

**Table 1:** Physical Characterization of Head-only RSV F Immunogens.

Construct	Oligomeric State^a^	Yield^b^ (mg/L)	Antibody affinity K_D_ (nM)			Physical Stability^c^							
						Temperature (°C)			pH		Osmolarity (mM)		Freeze-thaw
			D25	AM22	Mota^d^	50	70	90	3.5	10	10	3000	
DS-Cav1^e^	Trimer	1.9	0.15	<0.01	0.04	0.9	0.0	ND	0.8	0.9	1.0	0.8	0.7
i-273	Monomer	0.3	33.3	13.6	1360	1.0	1.1	1.3	1.0	1.0	0.8	0.9	1.1
i-693^f^	Dimer	0.5	9.9	20.3	6.5	0.8	1.2	0.1	0.8	0.8	1.4	1.4	0.6
i-210	Trimer	5.5	0.7	4.2	761	0.9	1.0	0.2	0.9	0.9	1.3	1.2	0.1
i-447	Trimer	3.6	0.7	1.2	0.5	1.0	1.0	0.1	1.0	1.0	1.0	0.5	0.5

^a^ Assessed by gel filtration.

^b^ Yield following gel filtration.

^c^ Fractional D25 reactivity after 60 minutes at specified conditions or 10 cycles of freeze-thaw.

^d^ Mota, motavizumab.

^e^ Values from reference [16].

^f^ Maximum initial fractional D25 reactivity is reported for physical stability.

ND, not determined.

We further assessed the stability of the purified immunogens by quantifying the effect of high temperature, pH extremes, osmolarity extremes, and cycles of freeze-thaw on recognition by antibody D25 (Table 1). All four immunogens were more thermostable than the RSV F DS-Cav1 trimer, and most were observed to be as robust as DS-Cav1 to other physical extremes. The only outliers were loss of recognition by D25 for i-210 after 10 freeze-thaw cycles and sensitivity of i-447 to high salt. Remarkably, the i-273 monomer was still viable after heating to 90°C for one hour and showed no loss of site Ø antigenicity after 10 cycles of freeze-thaw.

### Electron microscopy of top scoring head-only immunogens

**Fig 3:**
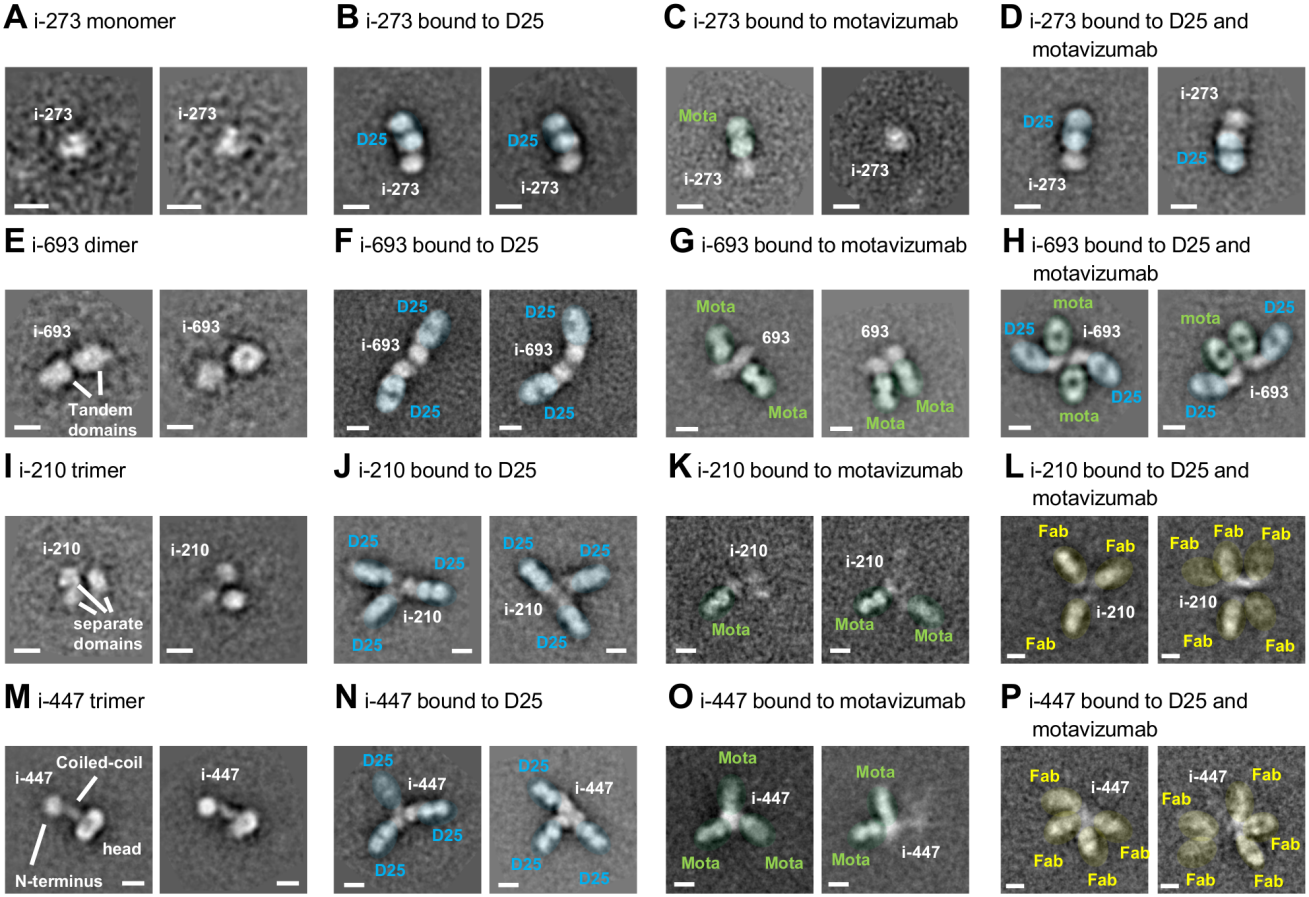
Negative-stain electron microscopy of head-only immunogens. Panels **(A-P)** show typical 2D averaged classes of i-273,i-693, i-210 and i-447 alone **(A, E, I** and **M)**, in complex with D25 (in blue; **B, F, J** and **N**), in complex with motavizumab (in green; **C, G, K** and **O**) and in complex with both D25 and motavizumab (in blue, green; **D, H, L** and **P**). Yellow indicates Fabs of ambiguous identity. Two separate averages are shown for each panel. White scale bars are 50 Å long.

All four immunogens were examined by negative-stain electron microscopy (EM) with and without antigen-binding fragments (Fabs) of antibodies D25 and motavizumab, followed by reference-free 2D classification and averaging to probe their structures (Figs 3 and S4). Immunogen i-273 was monomeric in structure (Figs 3A and S4A), and 1:1 binding to D25 Fabs was readily observed (Figs 3B, 3D, S4B and S4D). In contrast, binding to motavizumab alone was observed in less than 10% of the particles and i-273 was less distinct in these complexes (Figs 3C and S4C), consistent with its micromolar affinity to motavizumab. Furthermore, when i-273 was mixed with both D25 and motavizumab, only one bound Fab was observed in most cases (Figs 3D and S4D).

The i-693 tandemly linked dimer displayed a bi-lobed structure, with both lobes in close contact (Figs 3E and S4E). D25 bound to the distal ends of each lobe, suggesting the domains of i-693 to associate with each other through their membrane-proximal ends (Figs 3F, 3H, S4F and S4H). As expected from the structure of pre-fusion RSV F [9, 38], motavizumab Fabs bound at a different site, with an orientation rotated approximately 90° from D25 (Figs 3G, 3H, S4G and S4H). Differing orientations of the bound Fabs relative to each other were also observed, suggesting the linkage between the two domains to be quite flexible, and unlike immunogen i-273, i-693 readily bound both D25 and motavizumab Fabs simultaneously (Figs 3F–3H and S4F–S4H).

The i-210 trimer clearly displayed three lobes in many images (Figs 3I and S4I). However, the i-210 trimers were often heterogeneous in their quaternary arrangement with one or more domains appearing blurry, with greater separation between domains than might be expected, or with a tendency to form oligomers. Complexes with D25 had clear 1:3 stoichiometry, but the Fabs frequently appeared more distinct than the i-210 trimer, consistent with the heterogeneity noted above (Figs 3J and S4J). Similar to i-273 and consistent with the low micromolar affinity of i-210 for motavizumab, the i-210 trimer was observed to form more 1:1 or 1:2 complexes than 1:3 complexes with motavizumab (Figs 3K and S4K). Likewise, i-210 mixed with both D25 and motavizumab revealed mostly 1:3 complexes likely representing i-210 bound to D25 alone (Figs 3L and S3L).

The i-447 trimer displayed a dumb-bell shaped structure, with two differently sized lobes connected by a thin rod (Figs 3M and S4M). We interpreted the smaller lobe to be the frayed N-terminus of the rod-like coiled-coil and the larger lobe to be the trimeric RSV F head. Addition of Fab D25 resulted in trimeric star-like structures, consistent with a 1:3 binding stoichiometry and our proposed i-447 quaternary arrangement (Figs 3N and S4N). Deviations from perfect three-fold symmetry indicated some trimeric flexibility, though not to the extent observed for i-210. Binding of Fab motavizumab also resulted in trimeric star-like structures, with multiple examples of two clearly defined Fabs and one blurry Fab bound to i-447 (Figs 3O and S4O). These results suggested either this configuration to be not easily viewed in two dimensions or one protomer to have additional flexibility upon motavizumab Fab binding. Similarly, complexes of i-447 with both D25 and motavizumab resulted in star-like structures with 3–5 Fab arms, which were clearly present, though often blurry (Figs 3P and S4P).

### Immunogenicity of top scoring head-only immunogens

**Fig 4:**
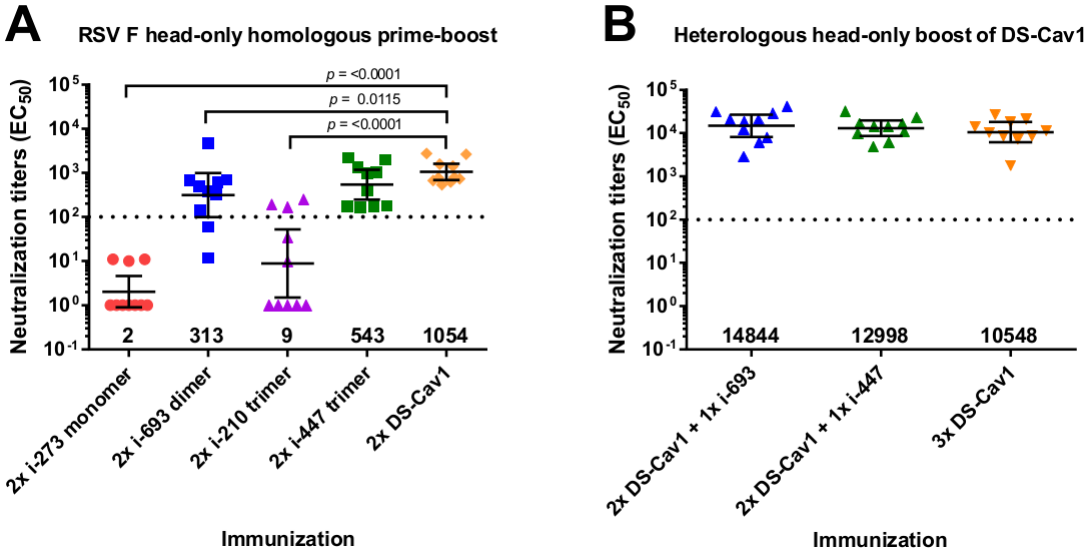
Head-only RSV F immunogen boost elicits neutralization titers surpassing DS-Cav1. **(A)** All four immunogens were used to immunize mice two times, resulting in high neutralization titers for i-693 and i-447 above the protective threshold of 100 (dotted line). *P* values between i-447 and i-210 or i-273 were 0.001 and <0.0001 respectively and those between i-693 and i-210 or i-273 were 0.0014 and <0.0001 respectively. **(B)** Immunogens i-693 and i-447 were used to boost DS-Cav1 seven weeks later resulting in titers higher than a DS-Cav1 homologous boost. Scatter plots show the geometric mean (numerical value below) with error bars representing the 95% confidence level. *P* values were determined by two-tailed Mann-Whitney tests. Each group included 10 mice.

To evaluate immunogenicity, each of the four top scoring head-only immunogens was used to immunize groups of 10 CB6F1/J mice. For each, 10 μg of the head-only immunogen, adjuvanted with 50 μg of polyinosinic:polycytidylic acid (polyI:C) was injected intramuscularly twice at an interval of three weeks. In week five sera, significantly higher reciprocal EC_50_ neutralization titers were observed in mice immunized with i-693 and i-447 than for i-210 and i-273 (GMT = 313, 543, 9 and 2 respectively, Fig 4A). Titers for i-693 were three-fold lower than that observed for the DS-Cav1 control; while titers for i-447 were statistically comparable to those induced by DS-Cav1.

The low neutralization titers for i-273 and i-210 were unexpected in light of the nanomolar affinity observed for the antigenic site Ø antibodies D25 and AM22 (Table 1 and S3 Fig). With the i-273 monomer, its relatively small size of 31 kDa may have hindered its immunogenicity [41] and its 13 hydrophilic surface mutations covering approximately 9% of the surface area (S2 Fig) may have distracted the antibody response from neutralizing epitopes. With the i-210 trimer, the quaternary heterogeneity observed by negative-stain EM suggested that unexpected neoepitopes may also be present, potentially diluting the B cell response to epitopes for neutralizing antibodies.

As immunogens i-693 and i-447 were both able to induce reasonable RSV-neutralizing antibodies, we further investigated their potential to focus the antibody response to head-only antigenic sites by heterologously boosting mice that had been primed twice with DS-Cav1. In each instance, the prime interval was three weeks and the boost interval was seven weeks. Such a boost may mimic the boosting of individuals who have been previously exposed to RSV. Notably, week 12 sera showed head-only boosting to elicit geometric mean titers for i-693 and i-447 of 14,844 and 12,998, respectively, which were statistically comparable with the mean titers for the RSV F DS-Cav1 control of 10,548 (Fig 4B). Interestingly, reversing the prime-boost (head-only prime followed by a DS-Cav1 boost) resulted in a significantly lower responses for both i-693 and i-447 (*P* = 0.0052 and 0.0354 respectively) (S5 Fig). Thus the dimeric i-693 and the trimeric i-447 did not appear to prime as well as the full length DS-Cav1, but they did appear to focus and to boost the head-directed neutralizing response.

### Head-only boosting can focus the RSV-neutralizing response to antigenic sites Ø and II

**Fig 5:**
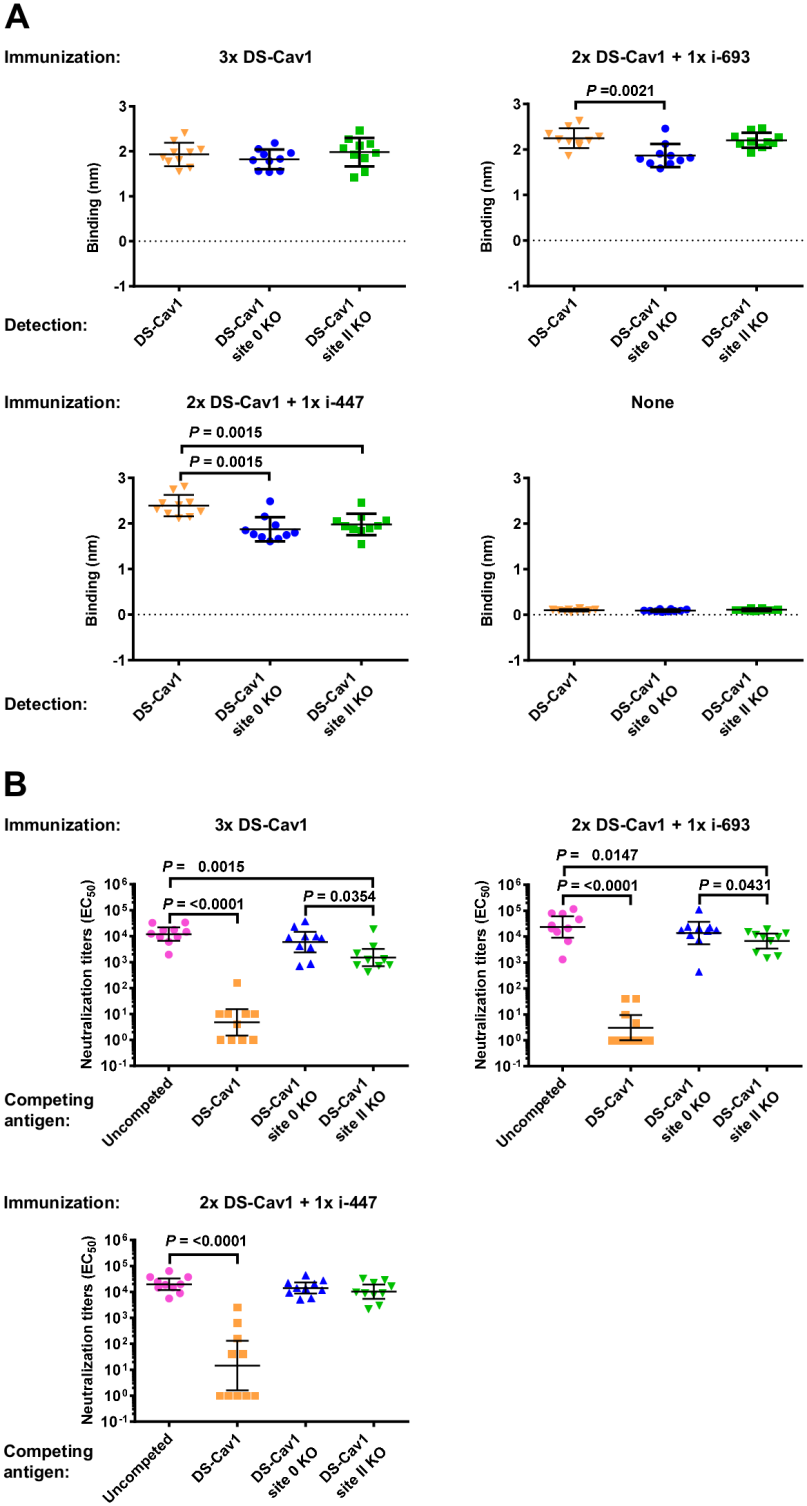
Boost by head-only RSV F immunogen can focus the RSV-neutralizing response to antigenic sites Ø and II. **(A)** Recognition of DS-Cav1, DS-Cav1 site Ø knockout (KO) and DS-Cav1 site II KO probes by sera from 2×DS-Cav1 immunized mice boosted with DS-Cav1, i-447 or i-693, respectively or unimmunized mice. **(B)** Neutralization competition of sera from 2×DS-Cav1 immunized mice boosted with DS-Cav1, i-447 or i-693, respectively. *P* values for differences between DS-Cav1 and DS-Cav1 site Ø or site II KO probes were all <0.0001. *P* values were determined by two-tailed Mann-Whitney test.

We next investigated whether the increased RSV-neutralization titers from the heterologous boosts of i-693 and i-447 were directed towards antigenic sites Ø or II. We previously developed DS-Cav1 probes with knock-out (KO) mutations in antigenic site Ø or antigenic site II [15], and we used these site-specific KO probes to quantify the site-specific immune response. First, we measured the relative binding affinity of immune sera to DS-Cav1, DS-Cav1 site Ø-KO, and DS-Cav1 site II-KO probes. With the homologous DS-Cav1 prime/boost sera, no significant difference was observed in binding to the three probes (Fig 5A). This was not entirely surprising given that the individual knock-out mutations for antigenic sites Ø and II cover only a small percentage (1–3%) of the surface area of their respective probes. In contrast, the site Ø-KO probe bound significantly less to both i-447 and i-693 boosted sera (*P* = 0.0015 and 0.0021 respectively) than to the DS-Cav1 probe (Fig 5A). The site II-KO probe also bound significantly less to i-447 boosted sera than to the DS-Cav1 probe (*P* = 0.0015), but its binding was statistically comparable to DS-Cav1 with i-693 boosted sera.

We next measured the competitive effect of DS-Cav1 antigenic site Ø and site II KO probes on neutralization. DS-Cav1 added to sera from each of the three groups of immunized mice (3×DS-Cav1, 2×DS-Cav1 + 1×i-693 and 2×DS-Cav1 + 1×i-447) competed for sera very efficiently as expected, resulting in significantly reduced neutralization (*P* = <0.0001) (Fig 5B). However, the site Ø KO and site II KO probes competed much less effectively than DS-Cav1, highlighting the importance of these sites (Fig 5B). In the DS-Cav1 and i-693-boosted group, antigenic site II KO probe competed more strongly than the antigenic site Ø KO probe (*P* = 0.0354 and 0.0431 respectively), indicating antigenic site Ø to be the dominant target of neutralization by these sera. In the i-447-boosted group, however, there was no significant difference in neutralization competition between antigenic site Ø and site II KO probes. Thus, with regards to site-specific induced responses, our results indicate the RSV-neutralizing response boosted by head-only immunogens i-693 and i-447 to indeed be more focused on head-specific sites Ø and II, especially antigenic site Ø.

## Discussion

A major goal for RSV-vaccine development is to identify immunogens that can boost antibody responses to neutralization-sensitive epitopes in the setting of pre-existing immunity. Increasing serum-neutralizing activity in pregnant women would help to increase their protective response and to extend the period of maternally-acquired protection from RSV. Likewise, in elderly people who have experienced repeated RSV infections throughout life, but still have modest levels of serum-neutralizing activity, boosting the response to neutralization-sensitive epitopes could achieve greater than threshold levels of protective immunity. Therefore, we took a structure-based design approach to produce immunogens that focused immune recognition on the apical surfaces of the pre-F trimer. We removed the stalk portion from the RSV F trimeric glycoprotein to create 70 RSV F head-only immunogens. Fifty of these designs were recognized by antibodies to the metastable antigenic site Ø (D25, AM22 and 5C4) and were stable for at least one hour at 70°C, a temperature at which DS-Cav1 rapidly inactivates (Table 1). The thermostable head-only immunogens included 26 monomers, 6 dimers, and 18 trimers. Four of these, a monomer (i-273), a dimer (i-693) and two trimers (i-210 and i-447), were expressed, purified, and characterized for antigenicity, overall stability, structural integrity, and immunogenicity. Although all four of these head-only immunogens displayed nanomolar affinity to antigenic site Ø-directed antibodies and had physical stability that was similar or exceeded that of DS-Cav1, only one of the immunogens (the i-447 trimer) was able to elicit RSV-neutralizing titers in mice that were comparable to those elicited by DS-Cav1 (Fig 4A). The lower overall immunogenicity of these head-only immunogens may relate to increased flexibility between protomers, as we recently found that the reduction of interprotomer flexibility in pre-F trimers yielded higher immunogenicity [42].

The two immunogens (i-273 and i-210) that elicited the lowest titers of RSV neutralization (geometric means of 2 and 9, respectively) had low micromolar affinity to the antigenic site II-directed antibody motavizumab, whereas i-693 and i-447 which induce substantially higher titers bound motavizumab at nanomolar affinities. This suggests that antigenic site II may potentially play a role in the overall effectiveness of the head-only immunogens, which is consistent with the neutralization competition data (Fig 5B). The small size and extensive surface mutagenesis of the i-273 monomer, and the greater structural heterogeneity and surface mutations of the i-210 trimer relative to i-447 may also have diminished the capabilities of these head-only immunogens.

By contrast, mice primed with DS-Cav1 and boosted with i-693 or i-447, elicited RSV-neutralizing responses that were statistically comparable to those boosted with DS-Cav1. Importantly, focusing of overall antibody response to both antigenic sites Ø and II was enhanced in i-693 and i-447-boosted mice relative to a DS-Cav1 boost (Fig 5A). Despite the smaller size of i-693 relative to i-447, the dimeric nature and flexibility of i-693 may have facilitated divalent antibody binding and thus enhanced boosting by i-693.

Our results demonstrate that a head-only RSV F immunogen boost can both focus and enhance the elicited response to antigenic sites associated with the RSV F head. The reduced size and increased thermostability of the head-only immunogens described here may also have advantages in terms of both expression and manufacturing. In addition to mammalian cell culture, these small immunogens may also be amenable to less expensive plant [43, 44] or bacterial-based expression systems [45, 46] as well as yeast [47, 48] or insect cell culture [49, 50]. Indeed, full length RSV F protein has been successfully expressed in apples [51], tomatoes [52], and insect cells [53]. Furthermore, the successful use of live chimeric vectors such as Sendai [54] or human parainfluenza virus type 3 [55] or replication-defective vectors such as adenovirus [56, 57] or Modified Vaccinia Ankara [57] to express RSV F protein and elicit immune responses in vector-immunized animals suggests alternative delivery methods could also be applied to head-only RSV F immunogens in a vaccination setting.

The design of even smaller RSV F head-derived immunogens with less extraneous non-neutralizing epitopes could potentially focus the RSV F immune response still further. However, such immunogen designs could be complicated if both non-proximal antigenic sites Ø and II were retained or if increased T cell help from the addition of universal T cell epitope peptides [58, 59] is required for the smallest immunogens. Although we did not observe a consistent correlation between oligomer size (i.e. monomer, dimer and trimer) and neutralization titers or immune focusing, increasing the immunogen oligomerization state in an ordered manner might also be expected to improve immune focusing since it would provide more opportunities for irrelevant epitopes to be masked by neighbor-neighbor interactions and the additional multivalency of the target epitope could lead to increased B cell avidity through antibody cross-linking, resulting in increased immunogenicity for the epitope [41, 60]. Therefore, additional multimerization by incorporation into nanoparticles [53, 61] might further improve both immune focusing and overall immunogenicity of the head-only RSV F immunogens.

It remains to be seen how many of the head-only immunogens are viable vaccine candidates. Results from the “best” four immunogens, nonetheless, already demonstrate the ability of head-only RSV F immunogens to boost to high titers previously elicited RSV-neutralizing responses to the RSV F glycoprotein. Together, these results indicate the head-only immunogens, i-693 and i-447, to be particularly suited to boosting RSV F-specific antibody responses in the setting of pre-existing immunity—a result of particular significance for maternal immunization and for vaccine protection of the elderly.

## Supporting Information

S1 ARRIVE Checklist Animal Research: Reporting In Vivo Experiments (ARRIVE) guidelines checklist. Page referencing of key issues relating to the animal work performed in this study.
(PDF)

S1 Fig Circular permutation of RSV F domain III. The left panel shows a ribbon diagram of domain III consisting of polypeptides F_1_ (green) and F_2_ (blue). Antigenic site Ø and site II are red and orange respectively. The middle and right panels depict two separate ways to reconnect F_1_ and F_2_ into a single chain through the addition of linkers (dotted lines). A cartoon of the genetic construct is shown above each ribbon diagram depicting the topology of the F_1_ (green) and F_2_ (blue) polypeptides.
(TIF)

S2 Fig Location of surface mutations on the i-273 and i-210 immunogens. For each immunogen a cartoon of the genetic construct is depicted (top) and a ribbon diagram (bottom), color-coded as in Figs 1 and S1. Surface mutations are depicted by magenta vertical lines in the genetic constructs and magenta spheres in the models.
(TIF)

S3 Fig Antigenicity of head-only immunogens. Biolayer interferometry sensorgrams for immunogens i-273, i-693, i-210 and i-447 respectively binding to D25 (left panels), AM22 (middle panels) and motavizumab (right panels). Each experiment employed a series of two-fold dilutions and the highest starting concentration is noted for each panel. The black lines represent the best fit of the kinetic data to a 1:1 binding model.
(TIF)

S4 Fig Negative-stain electron microscopy of head-only immunogens. Panels **(A-P)** show raw electron microscopy images with insets of collections of 2D averaged classes for i-273, i-693, i-210 and i-447 alone (**A, E, I** and **M**), in complex with D25 (**B, F, J** and **N**), in complex with motavizumab (**C, G, K** and **O**) and in complex with both D25 and motavizumab (**D, H, L** and **P**).
(TIF)

S5 Fig Neutralization titers from head-only RSV F immunogen primes boosted by DS-Cav1. Mice primed twice with i-693 or i-447 and boosted by DS-Cav1 at week 10 resulted in neutralization titers above the protective threshold of 100 (dotted line), but lower than a DS-Cav1 homologous boost. Scatter plots show the geometric mean (numerical value below) with error bars representing the 95% confidence level. *P* values between the 2× i-693 + 1× DS-Cav1 and 2× DS-Cav1 + 1× i-693 (Fig 4B) titers and between the 2× i-447 + 1× DS-Cav1 and 2× DS-Cav1 + 1× i-447 (Fig 4B) titers were 0.0052 and 0.0354 respectively. *P* values were determined by two-tailed Mann-Whitney tests. Each group included 10 mice.
(TIF)

S1 Table Amino acid sequences of head-only RSV F immunogens. (XLSX)

S2 Table Antigenic properties of monomeric head-only RSV F immunogens. (DOCX)

S3 Table Antigenic properties of dimeric head-only RSV F immunogens. (DOCX)

S4 Table Antigenic properties of trimeric head-only RSV F immunogens. (DOCX)
