# Supplementary figures and images for: Structure-Based Design of Head-Only Fusion Glycoprotein Immunogens for Respiratory Syncytial Virus
Source: PLoS One. 2016 Jul 27;11(7):e0159709. doi: 10.1371/journal.pone.0159709 (PMC4963090; doi:10.1371/journal.pone.0159709)

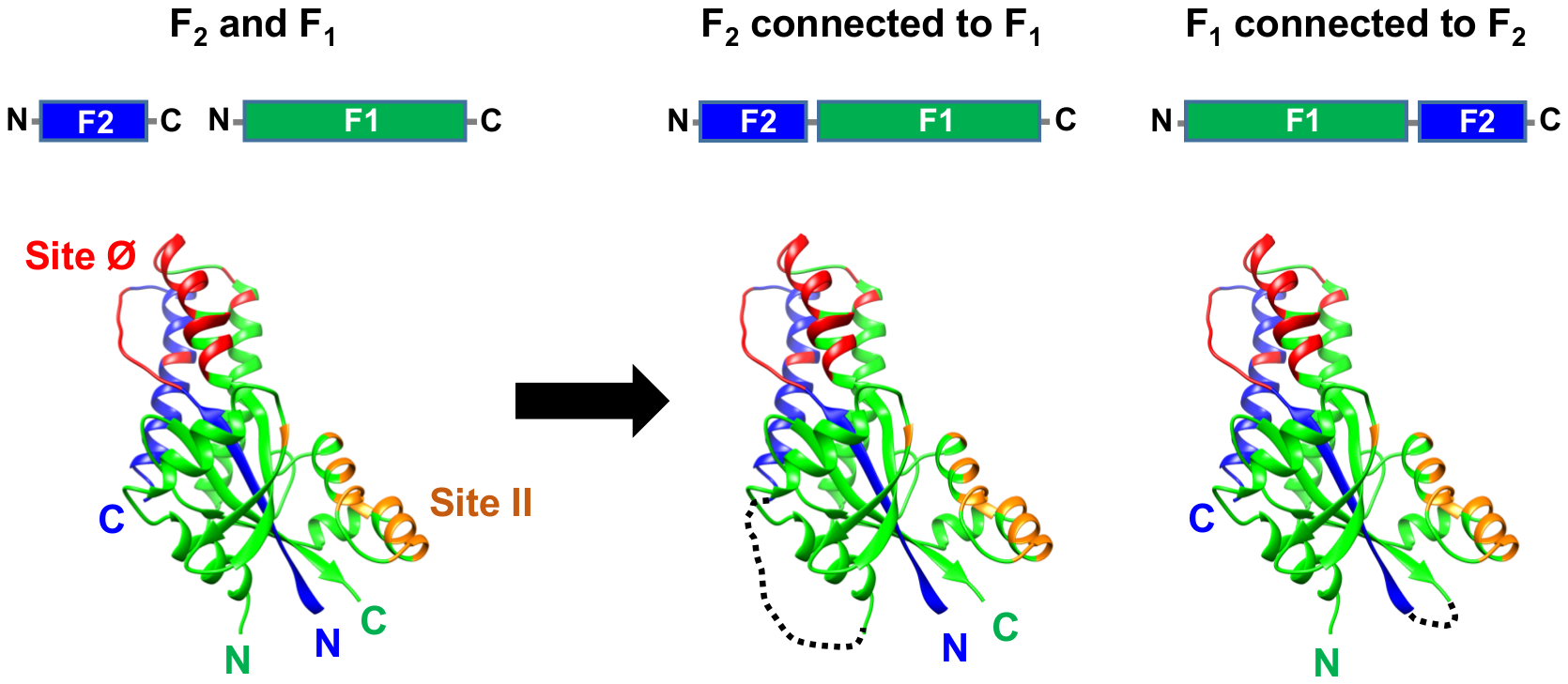

Supplement: S1 Fig — The left panel shows a ribbon diagram of domain III consisting of polypeptides F1 (green) and F2 (blue). Antigenic site Ø and site II are red and orange respectively. The middle and right panels depict two separate ways to reconnect F1 and F2 into a single chain through the addition of linkers (dotted lines). A cartoon of the genetic construct is shown above each ribbon diagram depicting the topology of the F1 (green) and F2 (blue) polypeptides. (TIF) [file pone.0159709.s002.tif]

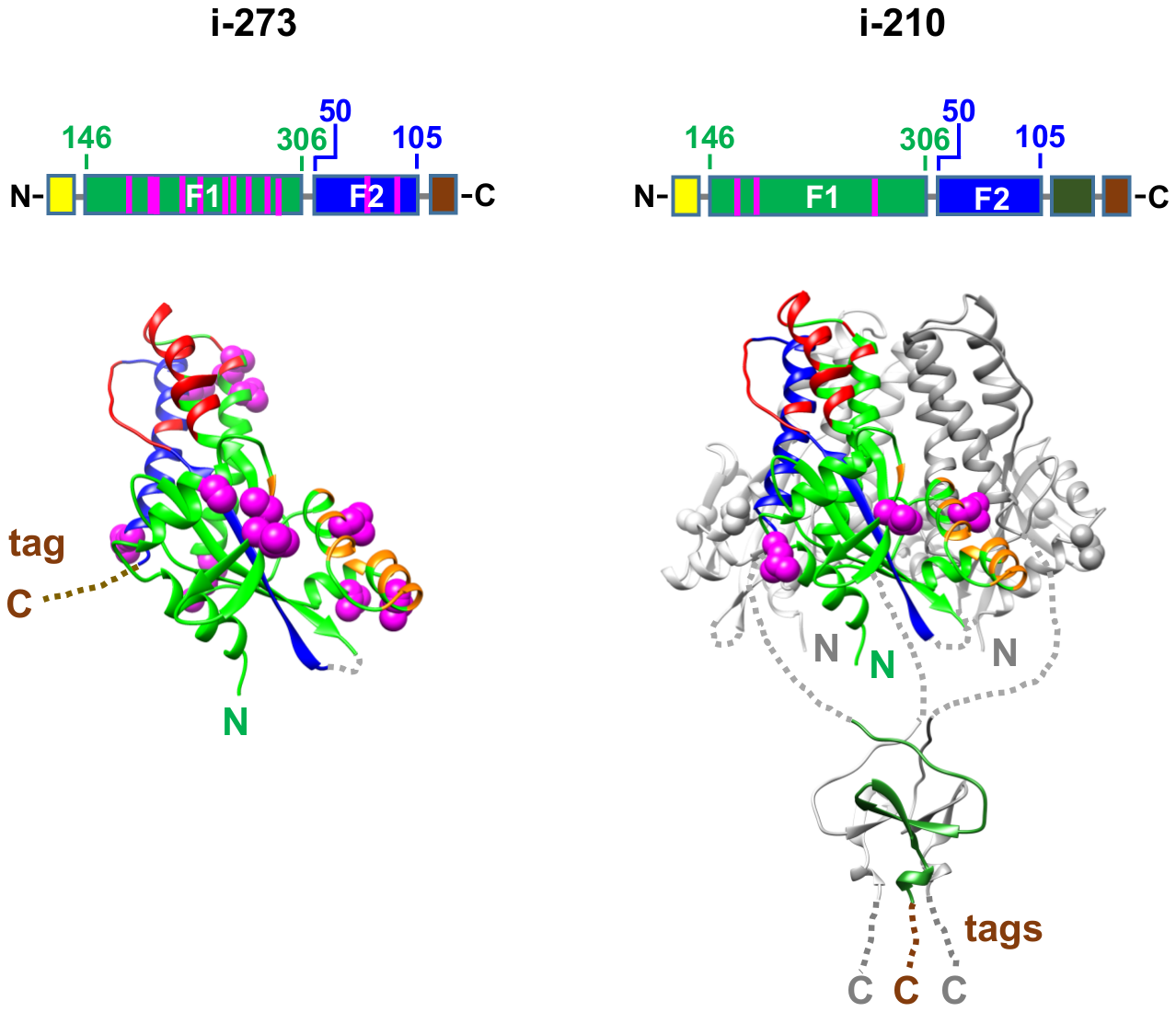

Supplement: S2 Fig — For each immunogen a cartoon of the genetic construct is depicted (top) and a ribbon diagram (bottom), color-coded as in Figs 1 and S1. Surface mutations are depicted by magenta vertical lines in the genetic constructs and magenta spheres in the models. (TIF) [file pone.0159709.s003.tif]

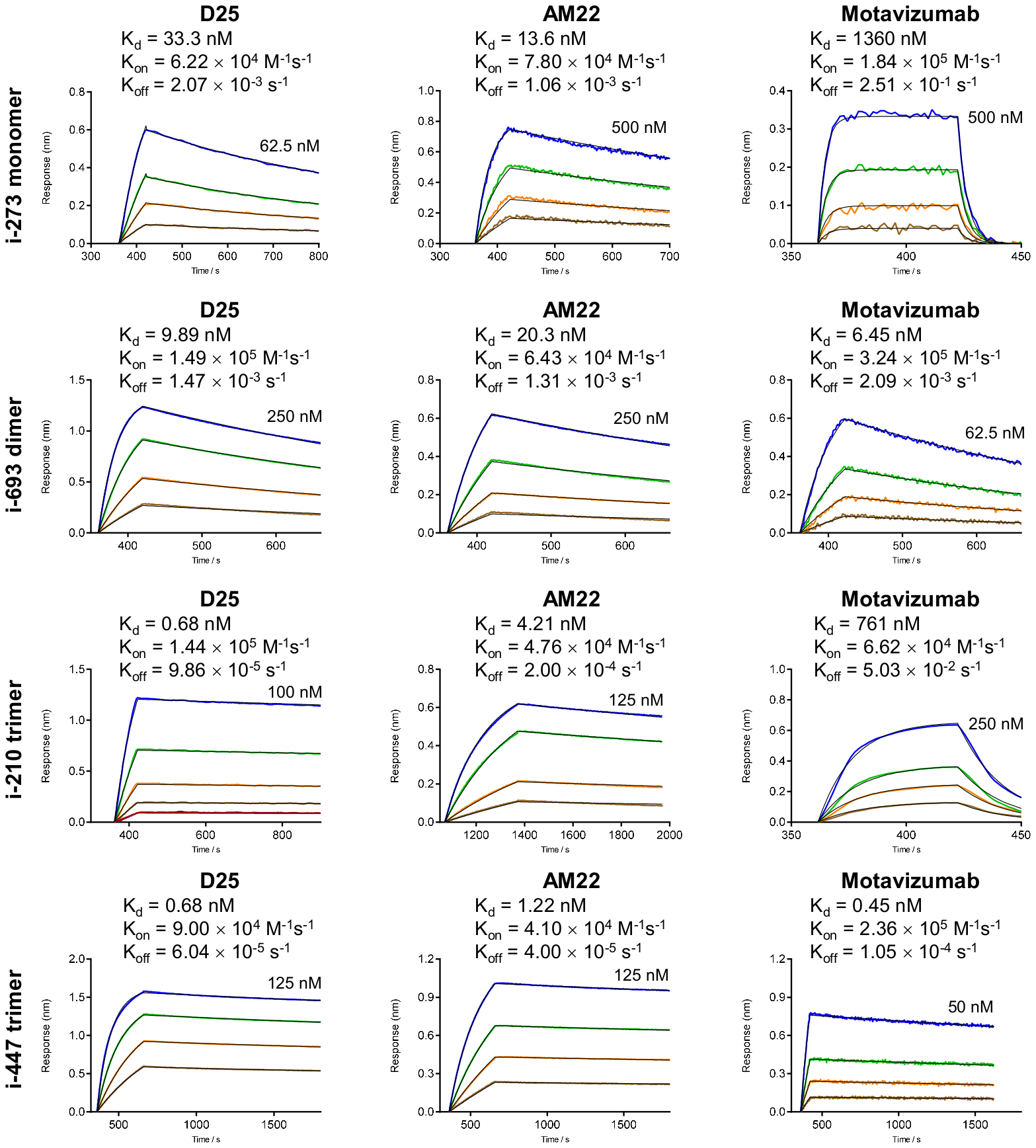

Supplement: S3 Fig — Biolayer interferometry sensorgrams for immunogens i-273, i-693, i-210 and i-447 respectively binding to D25 (left panels), AM22 (middle panels) and motavizumab (right panels). Each experiment employed a series of two-fold dilutions and the highest starting concentration is noted for each panel. The black lines represent the best fit of the kinetic data to a 1:1 binding model. (TIF) [file pone.0159709.s004.tif]

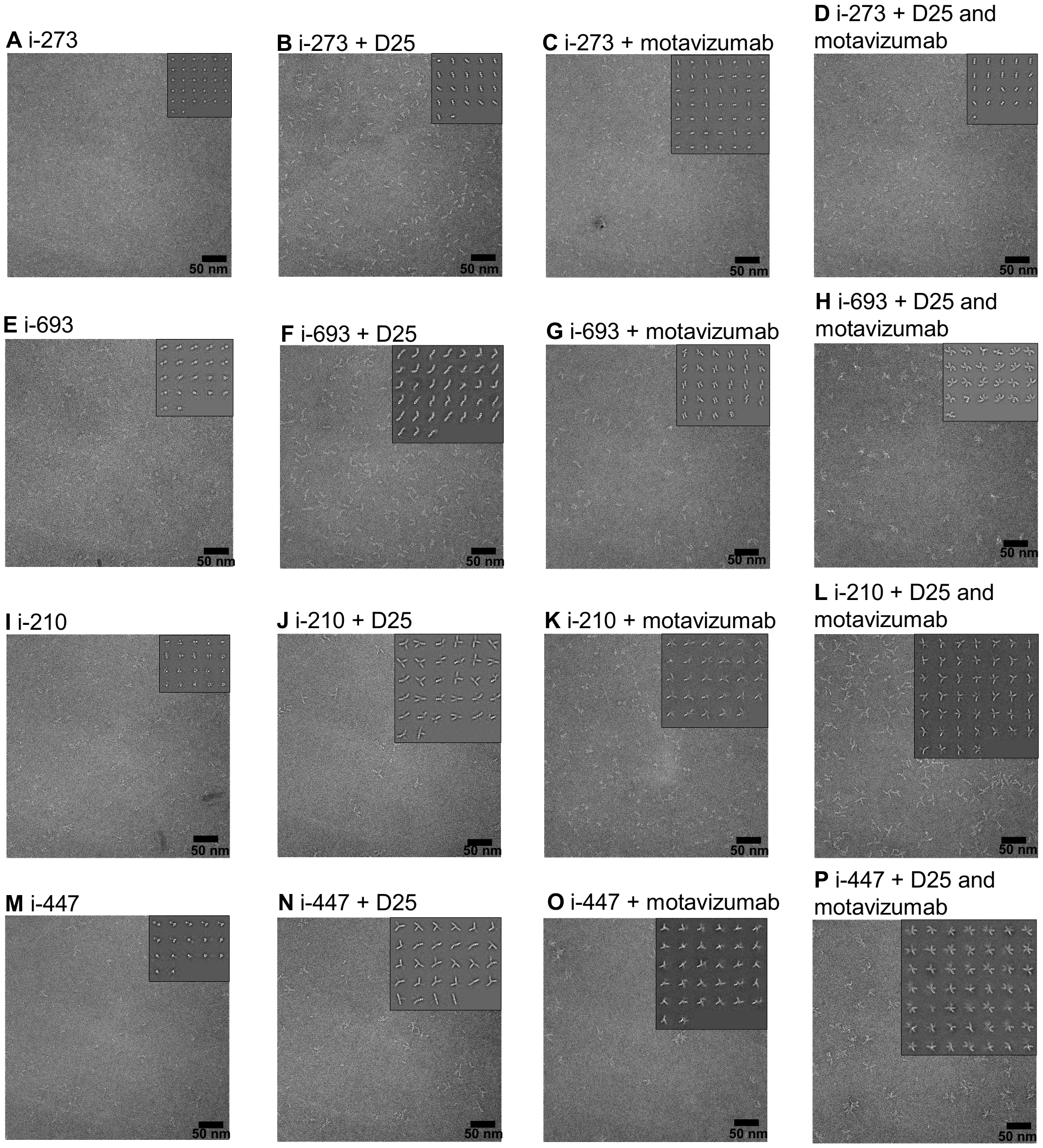

Supplement: S4 Fig — Panels (A-P) show raw electron microscopy images with insets of collections of 2D averaged classes for i-273, i-693, i-210 and i-447 alone (A, E, I and M), in complex with D25 (B, F, J and N), in complex with motavizumab (C, G, K and O) and in complex with both D25 and motavizumab (D, H, L and P). (TIF) [file pone.0159709.s005.tif]

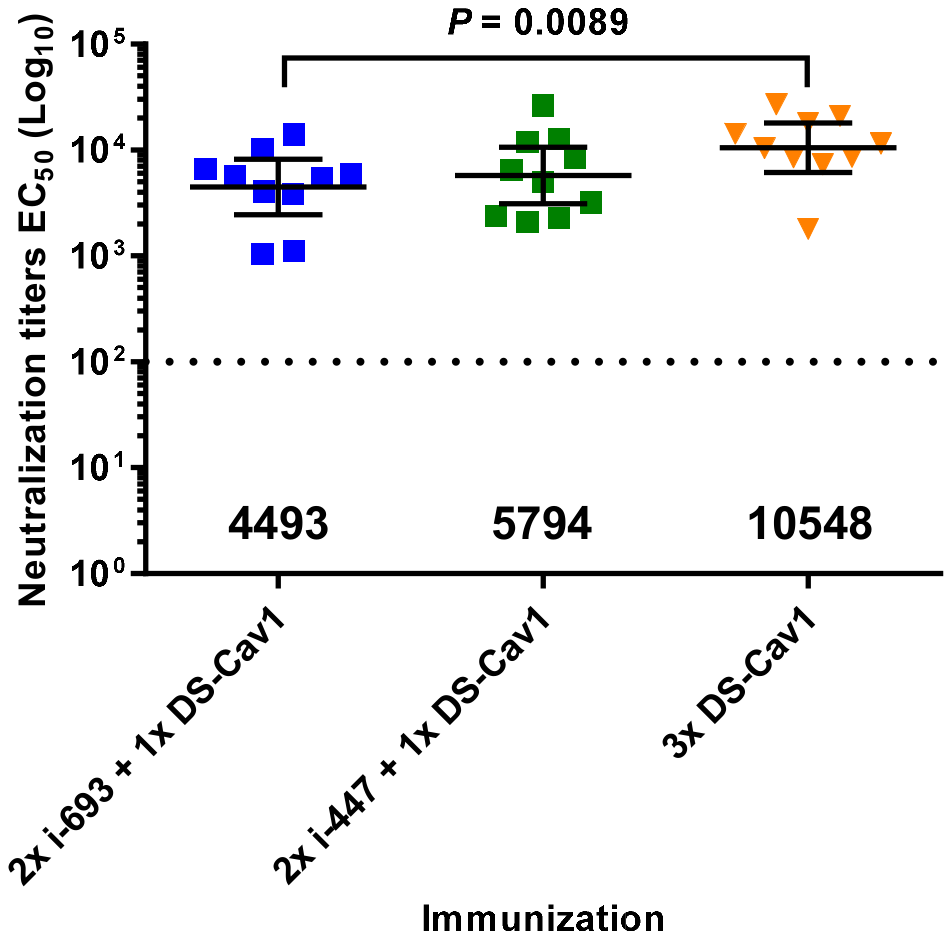

Supplement: S5 Fig — Mice primed twice with i-693 or i-447 and boosted by DS-Cav1 at week 10 resulted in neutralization titers above the protective threshold of 100 (dotted line), but lower than a DS-Cav1 homologous boost. Scatter plots show the geometric mean (numerical value below) with error bars representing the 95% confidence level. P values between the 2× i-693 + 1× DS-Cav1 and 2× DS-Cav1 + 1× i-693 (Fig 4B) titers and between the 2× i-447 + 1× DS-Cav1 and 2× DS-Cav1 + 1× i-447 (Fig 4B) titers were 0.0052 and 0.0354 respectively. P values were determined by two-tailed Mann-Whitney tests. Each group included 10 mice. (TIF) [file pone.0159709.s006.tif]
