# Supplementary material for: Structure-Based Design of Head-Only Fusion Glycoprotein Immunogens for Respiratory Syncytial Virus
Source: PLoS One. 2016 Jul 27;11(7):e0159709. doi: 10.1371/journal.pone.0159709 (PMC4963090; doi:10.1371/journal.pone.0159709)
Supplement: S2 Table — (DOCX) [file pone.0159709.s008.docx]

**S2 Table. Antigenic properties of monomeric head-only RSV F immunogens**

| **Design** | **D25 binding^a^ upon expression** | **D25 binding^a^ after 1 week at 4°C** | **D25 binding^a^ after 1 hour at elevated temperature** | | | | | **5C4^b^ binding^a^** | **AM22**^c^ **binding^a^** | **Average^d^ D25 and AM22 binding^a^** |
| --- | --- | --- | --- | --- | --- | --- | --- | --- | --- | --- |
|  |  |  | **60°C** | **70°C** | **80°C** | **90°C** | **100°C** |  |  |  |
| **Controls** | | | | | | | | | | |
| DS-Cav1 | 1.77 | 2.40 | 1.33 | 0.06 | 0.05 | 0.06 | ND | 2.64 | 2.80 | 1.75 |
| RSV F Fd | 1.93 | 0.15 | 0.18 | 0.05 | 0.06 | 0.09 | ND | ND | 0.92 | 0.37 |
| **Domain III monomer (30 total)** | | | | | | | | | | |
| i-66 | 2.03 | 2.06 | 2.06 | 1.87 | 0.11 | 0.06 | ND | 2.87 | 2.16 | 2.03 |
| i-69 | 0.12 | 0.29 | 0.16 | 0.09 | 0.08 | 0.05 | ND | 1.53 | 1.22 | 0.54 |
| i-186 | 2.56 | 2.54 | 2.33 | 2.56 | 0.22 | 0.06 | ND | 2.67 | 3.11 | 2.74 |
| i-189 | 2.66 | 2.90 | 2.52 | 2.64 | 0.27 | 0.05 | ND | 2.74 | 3.07 | 2.87 |
| i-192 | 2.78 | 2.86 | 2.55 | 2.19 | 0.85 | 0.22 | ND | 2.47 | 3.13 | 2.73 |
| i-195 | 2.85 | 3.09 | 3.07 | 2.75 | 0.40 | 0.14 | ND | 3.06 | 3.11 | 2.98 |
| i-198 | 2.79 | 2.64 | 2.75 | 2.57 | 1.77 | 0.05 | ND | 3.05 | 3.11 | 2.77 |
| i-201 | 3.11 | 3.19 | 2.95 | 1.91 | 1.05 | 0.13 | ND | 3.08 | 3.23 | 2.78 |
| i-204 | 2.96 | 3.21 | 2.94 | 2.18 | 2.78 | 1.13 | 2.17 | 2.98 | 3.25 | 2.88 |
| i-234 | 2.84 | 2.90 | 2.81 | 2.58 | 0.74 | 0.38 | ND | 2.82 | 1.45 | 2.31 |
| i-237 | 2.79 | 2.90 | 2.61 | 2.55 | 0.52 | 0.28 | ND | 2.71 | 3.11 | 2.86 |
| i-240 | 2.79 | 2.86 | 2.50 | 1.86 | 0.48 | 0.08 | ND | 2.61 | 3.09 | 2.60 |
| i-243 | 2.81 | 3.03 | 3.02 | 2.78 | 0.98 | 0.07 | ND | 3.09 | 3.10 | 2.97 |
| i-246 | 2.78 | 2.51 | 2.72 | 2.33 | 1.49 | 0.06 | ND | 3.05 | 3.10 | 2.65 |
| i-249 | 3.14 | 3.17 | 3.05 | 2.71 | 0.83 | 0.23 | ND | 3.19 | 3.20 | 3.03 |
| i-252 | 3.08 | 3.16 | 3.03 | 2.59 | 1.30 | 0.22 | ND | 3.17 | 2.48 | 2.74 |
| i-270 | 2.86 | 3.06 | 3.03 | 2.74 | 1.52 | 0.20 | ND | 3.10 | 3.15 | 2.98 |
| i-273 | 3.09 | 3.20 | 3.09 | 3.08 | 3.03 | 0.74 | ND | 3.17 | 3.23 | 3.17 |
| i-276 | 3.03 | 3.25 | 3.16 | 2.81 | 1.07 | 0.20 | ND | 3.08 | 3.25 | 3.10 |
| i-279 | 1.81 | 1.02 | 0.95 | 0.21 | 0.37 | 0.09 | ND | 1.87 | 2.80 | 1.34 |
| i-282 | 2.63 | 2.82 | 2.61 | 2.87 | 1.69 | 0.14 | ND | 2.96 | 3.32 | 3.00 |
| i-342 | 2.08 | 2.84 | 2.13 | 0.08 | 1.53 | 0.11 | ND | 3.05 | 2.63 | 1.85 |
| i-303 | 2.87 | 3.20 | 3.18 | 3.20 | 2.83 | 0.80 | ND | 3.26 | 2.89 | 3.10 |
| i-306 | 2.71 | 3.16 | 2.99 | 3.16 | 2.85 | 0.20 | ND | 3.23 | 2.85 | 3.06 |
| i-309 | 2.32 | 3.10 | 2.81 | 3.10 | 1.30 | 0.25 | ND | 3.20 | 2.81 | 3.01 |
| i-312 | 0.51 | 2.81 | 2.14 | 2.81 | 1.78 | 0.13 | ND | 3.04 | 2.82 | 2.81 |
| i-672 | 2.54 | 1.29 | 2.08 | 1.29 | 0.04 | 0.09 | ND | 3.06 | 2.84 | 1.80 |
| i-675 | 2.48 | 3.06 | 3.02 | 3.06 | 0.12 | 0.09 | ND | 3.13 | 2.93 | 3.02 |
| i-678 | 2.37 | 1.76 | 2.87 | 1.76 | 0.07 | 0.07 | ND | 3.21 | 2.84 | 2.12 |
| i-681 | 2.55 | 2.94 | 3.09 | 2.94 | 0.17 | 0.07 | ND | 3.20 | 2.86 | 2.91 |
| **Total > 1.5^e^** | **28** | **27** | **28** | **26** | **9** | **0** | **1** | **30** | **28** | **28** |

**^a^** ELISA binding assessed by the optical density at 450 nm. Values are color-coded: white, 0.0-0.19; green, 0.20-0.49; yellow, 0.50-1.49; red, 1.50-4.00.

**^b^** 5C4 binding assessed after 5 weeks at 4°C.

^c^ AM22 binding assessed after 2 weeks at 4°C.

**^d^** Average of D25 binding after 1 week at 4°C, D25 binding after 1 hour at 70°C and AM22 binding after 2 weeks at 4°C.

^e^ Total designs for each column with ELISA values > 1.5

ND, not determined for 100°C when the ELISA reading for 90°C was less than 1.00.
