# Supplementary material for: Structure-Based Design of Head-Only Fusion Glycoprotein Immunogens for Respiratory Syncytial Virus
Source: PLoS One. 2016 Jul 27;11(7):e0159709. doi: 10.1371/journal.pone.0159709 (PMC4963090; doi:10.1371/journal.pone.0159709)
Supplement: S3 Table — (DOCX) [file pone.0159709.s009.docx]

**S3 Table. Antigenic properties of dimeric head-only RSV F immunogens**

| **Design** | **D25 binding^a^ upon expression** | **D25 binding^a^ after 1 week at 4°C** | **D25 binding^a^ after 1 hour at elevated temperature** | | | | | **5C4^b^ binding^a^** | **AM22**^c^ **binding^a^** | **Average^d^ D25 and AM22 binding^a^** |
| --- | --- | --- | --- | --- | --- | --- | --- | --- | --- | --- |
|  |  |  | **60°C** | **70°C** | **80°C** | **90°C** | **100°C** |  |  |  |
| **Tandem domain III (18 total)** | | | | | | | | | | |
| i-684 | 1.84 | 0.82 | 2.20 | 0.82 | 0.06 | 0.06 | ND | 3.15 | 2.85 | 1.50 |
| i-687 | 2.54 | 3.12 | 2.77 | 3.12 | 0.07 | 0.07 | ND | 3.28 | 2.86 | 3.04 |
| i-690 | 2.62 | 2.50 | 2.64 | 2.50 | 0.06 | 0.07 | ND | 3.21 | 2.90 | 2.64 |
| i-693 | 2.75 | 3.14 | 2.60 | 3.14 | 0.10 | 0.07 | ND | 3.19 | 2.90 | 3.06 |
| i-360 | 1.60 | 1.07 | 1.90 | 1.07 | 0.08 | 0.05 | ND | 3.01 | 2.70 | 1.61 |
| i-363 | 2.72 | 3.04 | 2.73 | 3.04 | 0.08 | 0.06 | ND | 3.17 | 2.79 | 2.96 |
| i-366 | 2.54 | 2.74 | 2.45 | 2.74 | 0.06 | 0.05 | ND | 3.14 | 2.76 | 2.75 |
| i-369 | 2.29 | 3.07 | 2.45 | 3.07 | 0.14 | 0.05 | ND | 3.16 | 2.72 | 2.95 |
| i-372 | 0.05 | 0.05 | 0.06 | 0.05 | 0.05 | 0.05 | ND | 0.14 | 0.44 | 0.18 |
| i-375 | 2.58 | 0.59 | 2.45 | 0.59 | 0.23 | 0.09 | ND | 3.15 | 2.70 | 1.29 |
| i-378 | 0.07 | 0.05 | 0.06 | 0.05 | 0.05 | 0.05 | ND | 0.15 | 0.24 | 0.11 |
| i-381 | 0.56 | 0.20 | 1.46 | 0.20 | 0.06 | 0.05 | ND | 2.80 | 2.53 | 0.97 |
| i-384 | 0.08 | 0.05 | 0.10 | 0.05 | 0.04 | 0.05 | ND | 0.21 | 0.30 | 0.13 |
| i-387 | 0.11 | 0.74 | 0.32 | 0.74 | 0.40 | 0.06 | ND | 1.28 | 2.12 | 1.20 |
| i-390 | 0.05 | 0.05 | 0.12 | 0.05 | 0.05 | 0.05 | ND | 0.12 | 0.16 | 0.09 |
| i-393 | 0.10 | 0.61 | 0.19 | 0.61 | 0.23 | 0.05 | ND | 0.93 | 1.55 | 0.92 |
| i-396 | 0.06 | 0.05 | 0.09 | 0.05 | 0.06 | 0.05 | ND | 0.14 | 0.35 | 0.15 |
| i-399 | 0.12 | 0.54 | 0.24 | 0.54 | 0.41 | 0.05 | ND | 1.52 | 1.91 | 1.00 |
| **Total > 1.5**^e^ | **9** | **6** | **9** | **6** | **0** | **0** | **0** | **11** | **13** | **7** |

**^a^** ELISA binding assessed by the optical density at 450 nm. Values are color-coded: white, 0.0-0.19; green, 0.20-0.49; yellow, 0.50-1.49; red, 1.50-4.00.

**^b^** 5C4 binding assessed after 5 weeks at 4°C.

^c^ AM22 binding assessed after 2 weeks at 4°C.

^d^ Average of D25 binding after 1 week at 4°C, D25 binding after 1 hour at 70°C and AM22 binding after 2 weeks at 4°C.

^e^ Total designs for each column with ELISA values > 1.5

ND, not determined for 100°C when the ELISA reading for 90°C was less than 1.00.
