# Supplementary material for: Structure-Based Design of Head-Only Fusion Glycoprotein Immunogens for Respiratory Syncytial Virus
Source: PLoS One. 2016 Jul 27;11(7):e0159709. doi: 10.1371/journal.pone.0159709 (PMC4963090; doi:10.1371/journal.pone.0159709)
Supplement: S4 Table — (DOCX) [file pone.0159709.s010.docx]

**S4 Table. Antigenic properties of trimeric head-only RSV F immunogens**

| **Design** | **D25 binding^a^ upon expression** | **D25 binding^a^ after 1 week at 4°C** | **D25 binding^a^ after 1 hour at elevated temperature** | | | | | **5C4^b^ binding^a^** | **AM22**^c^ **binding^a^** | **Average^d^ D25 and AM22 binding^a^** |
| --- | --- | --- | --- | --- | --- | --- | --- | --- | --- | --- |
|  |  |  | **60°C** | **70°C** | **80°C** | **90°C** | **100°C** |  |  |  |
| **Trimerized domain III (22 total)** | | | | | | | | | | |
| i-426 | 2.62 | 1.39 | 2.91 | 1.39 | 0.24 | 0.14 | ND | 3.22 | 2.79 | 1.86 |
| i-429 | 2.58 | 1.34 | 2.83 | 1.34 | 0.23 | 0.13 | ND | 3.20 | 2.71 | 1.80 |
| i-432 | 2.00 | 2.17 | 2.53 | 2.17 | 0.07 | 0.05 | ND | 3.09 | 2.73 | 2.36 |
| i-435 | 0.05 | 0.05 | 0.08 | 0.05 | 0.05 | 0.06 | ND | 0.39 | 0.77 | 0.29 |
| i-438 | 2.16 | 3.05 | 2.82 | 3.05 | 0.10 | 0.06 | ND | 3.14 | 2.76 | 2.95 |
| i-441 | 2.49 | 1.46 | 3.03 | 1.46 | 0.23 | 0.11 | ND | 3.25 | 2.73 | 1.89 |
| i-444 | 2.65 | 1.59 | 3.03 | 1.59 | 0.23 | 0.12 | ND | 3.29 | 2.75 | 1.97 |
| i-447 | 2.78 | 3.23 | 3.01 | 3.23 | 0.13 | 0.06 | ND | 3.28 | 2.79 | 3.08 |
| i-450 | 2.50 | 2.70 | 2.76 | 2.70 | 0.12 | 0.06 | ND | 3.25 | 2.74 | 2.72 |
| i-453 | 0.40 | 0.19 | 0.80 | 0.19 | 0.06 | 0.05 | ND | 2.49 | 2.38 | 0.92 |
| i-207 | 2.91 | 3.17 | 3.05 | 3.03 | 1.00 | 0.11 | ND | 2.81 | 2.40 | 2.87 |
| i-210 | 2.89 | 3.11 | 2.96 | 2.95 | 1.34 | 0.47 | ND | 2.85 | 3.17 | 3.08 |
| i-213 | 2.91 | 3.02 | 2.73 | 2.77 | 2.22 | 1.31 | 2.22 | 2.93 | 3.11 | 2.96 |
| i-216 | 2.87 | 3.03 | 2.70 | 2.95 | 0.71 | 0.18 | ND | 2.62 | 3.12 | 3.03 |
| i-219 | 2.96 | 3.06 | 3.03 | 2.95 | 1.20 | 0.12 | ND | 3.04 | 3.06 | 3.02 |
| i-255 | 2.98 | 3.14 | 3.01 | 2.91 | 0.41 | 0.06 | ND | 3.01 | 0.86 | 2.30 |
| i-258 | 2.89 | 3.05 | 2.86 | 2.75 | 0.38 | 0.06 | ND | 2.97 | 1.28 | 2.36 |
| i-261 | 2.83 | 3.09 | 2.74 | 2.08 | 0.49 | 0.14 | ND | 2.98 | 3.14 | 2.77 |
| i-264 | 2.91 | 2.93 | 2.70 | 2.59 | 0.28 | 0.06 | ND | 2.69 | 3.13 | 2.88 |
| i-267 | 2.93 | 3.08 | 3.06 | 2.95 | 1.77 | 0.07 | ND | 3.23 | 3.12 | 3.05 |
| i-708 | 2.88 | 0.10 | 3.16 | 0.10 | 0.07 | 0.06 | ND | 3.26 | 2.97 | 1.06 |
| i-711 | 2.84 | 2.75 | 3.10 | 2.75 | 0.10 | 0.07 | ND | 3.33 | 3.03 | 2.84 |
| i-696 | 0.61 | 0.08 | 0.82 | 0.08 | 0.04 | 0.09 | ND | 2.50 | 2.70 | 0.95 |
| i-699 | 1.35 | 2.46 | 2.24 | 2.46 | 0.06 | 0.08 | ND | 3.01 | 2.90 | 2.61 |
| i-702 | 1.21 | 0.73 | 2.30 | 0.73 | 0.06 | 0.07 | ND | 3.06 | 2.95 | 1.47 |
| i-705 | 2.49 | 3.04 | 2.99 | 3.04 | 0.08 | 0.07 | ND | 3.18 | 2.96 | 3.01 |
| i-714 | 2.32 | 0.81 | 2.59 | 0.81 | 0.07 | 0.07 | ND | 3.23 | 2.96 | 1.53 |
| i-345 | 2.61 | 1.47 | 2.73 | 0.06 | 0.06 | 0.05 | ND | 3.15 | 2.71 | 1.41 |
| i-348 | 0.28 | 0.06 | 0.35 | 0.06 | 0.07 | 0.05 | ND | 2.35 | 2.41 | 0.85 |
| i-351 | 0.46 | 0.10 | 0.96 | 0.10 | 0.06 | 0.05 | ND | 2.75 | 2.50 | 0.90 |
| i-354 | 0.26 | 0.07 | 2.00 | 0.07 | 0.06 | 0.06 | ND | 2.38 | 2.24 | 0.79 |
| i-357 | 0.50 | 0.19 | 0.83 | 0.19 | 0.05 | 0.05 | ND | 2.68 | 2.49 | 0.96 |
| **Total > 1.5**^e^ | **23** | **18** | **26** | **18** | **2** | **0** | **1** | **31** | **29** | **22** |

**^a^** ELISA binding assessed by the optical density at 450 nm. Values are color-coded: white, 0.0-0.19; green, 0.20-0.49; yellow, 0.50-1.49; red, 1.50-4.00.

**^b^** 5C4 binding assessed after 5 weeks at 4°C.

^c^ AM22 binding assessed after 2 weeks at 4°C.

**^d^** Average of D25 binding after 1 week at 4°C, D25 binding after 1 hour at 70°C and AM22 binding after 2 weeks at 4°C.

^e^ Total designs for each column with ELISA values > 1.5

ND, not determined for 100°C when the ELISA reading for 90°C was less than 1.00.
